# Gaussian Multiple Access Channels with One-Bit Quantizer at the Receiver †^,^‡

**DOI:** 10.3390/e20090686

**Published:** 2018-09-07

**Authors:** Borzoo Rassouli, Morteza Varasteh, Deniz Gündüz

**Affiliations:** 1 School of Computer Science and Electronic Engineering, University of Essex, Colchester CO4 3SQ, UK; 2 The Intelligent Systems and Networks group of Department of Electrical and Electronics, Imperial College London, London SW7 2AZ, UK

**Keywords:** Gaussian multiple access channel, one-bit quantizer, capacity region

## Abstract

The capacity region of a two-transmitter Gaussian multiple access channel (MAC) under average input power constraints is studied, when the receiver employs a zero-threshold one-bit analogue-to-digital converter (ADC). It is proven that the input distributions of the two transmitters that achieve the boundary points of the capacity region are discrete. Based on the position of a boundary point, upper bounds on the number of the mass points of the corresponding distributions are derived. Furthermore, a lower bound on the sum capacity is proposed that can be achieved by time division with power control. Finally, inspired by the numerical results, the proposed lower bound is conjectured to be tight.

## 1. Introduction

The energy consumption of an analogue-to-digital converter (ADC) (measured in Joules/sample) grows exponentially with its resolution (in bits/sample) [1,2].When the available power is limited, for example, for mobile devices with limited battery capacity, or for wireless receivers that operate on limited energy harvested from ambient sources [3], the receiver circuitry may be constrained to operate with low resolution ADCs. The presence of a low-resolution ADC, in particular a one-bit ADC at the receiver, alters the channel characteristics significantly. Such a constraint not only limits the fundamental bounds on the achievable rate, but it also changes the nature of the communication and modulation schemes approaching these bounds. For example, in a real additive white Gaussian noise (AWGN) channel under an average power constraint on the input, if the receiver is equipped with a *K*-bin (i.e., $\log_{2}K$-bit) ADC front end, it is shown in [4] that the capacity-achieving input distribution is discrete with at most K+1 mass points. This is in contrast with the optimality of the Gaussian input distribution when the receiver has infinite resolution.

Especially with the adoption of massive multiple-input multiple-output (MIMO) receivers and the millimetre wave (mmWave) technology enabling communication over large bandwidths, communication systems with limited-resolution receiver front ends are becoming of practical importance. Accordingly, there has been a growing research interest in understanding both the fundamental information theoretic limits and the design of practical communication protocols for systems with finite-resolution ADC front ends. In [5], the authors showed that for a Rayleigh fading channel with a one-bit ADC and perfect channel state information at the receiver (CSIR), quadrature phase shift keying (QPSK) modulation is capacity-achieving. In case of no CSIR, [6] showed that QPSK modulation is optimal when the signal-to-noise ratio (SNR) is above a certain threshold, which depends on the coherence time of the channel, while for SNRs below this threshold, on-off QPSK achieves the capacity. For the point-to-point multiple-input multiple-output (MIMO) channel with a one-bit ADC front end at each receive antenna and perfect CSIR, [7] showed that QPSK is optimal at very low SNRs, while with perfect channel state information at the transmitter (CSIT), upper and lower bounds on the capacity are provided in [8].

To the best of our knowledge, the existing literature on communications with low-resolution ADCs focuses exclusively on point-to-point systems. Our goal in this paper is to understand the impact of low-resolution ADCs on the capacity region of a multiple access channel (MAC). In particular, we consider a two-transmitter Gaussian MAC with a one-bit quantizer at the receiver. The inputs to the channel are subject to average power constraints. We show that any point on the boundary of the capacity region is achieved by discrete input distributions. Based on the slope of the tangent line to the capacity region at a boundary point, we propose upper bounds on the cardinality of the support of these distributions. Finally, based on numerical analysis for the sum capacity, it is observed that we cannot obtain a sum rate higher than is achieved by time division with power control.

The paper is organized as follows. Section 2 introduces the system model. In Section 3, the capacity region of a general two-transmitter memoryless MAC under input average power constraints is investigated. The main result of the paper is presented in Section 3, and a detailed proof is given in Section 4. The proof has two parts: (1) it is shown that the support of the optimal distributions is bounded by contradiction; and (2) we make use of this boundedness to prove the finiteness of the optimal support by using Dubins’ theorem [9]. Section 5 analyses the sum capacity, and finally, Section 6 concludes the paper.

Notations: Random variables are denoted by capital letters, while their realizations with lower case letters. $F_{X}\left(x\right)$ denotes the cumulative distribution function (CDF) of random variable *X*. The conditional probability mass function (pmf) $p_{Y|X_{1},X_{2}}\left(y|x_{1},x_{2}\right)$ will be written as $p(y|x_{1},x_{2})$. For integers m≤n, we have [m:n]={m,m+1,…,n}. For 0≤t≤1, $H_{b}\left(t\right)≜-t\log_{2}t-\left(1-t\right)\log_{2}\left(1-t\right)$ denotes the binary entropy function. The unit-step function is denoted by s(·).

## 2. System Model and Preliminaries

**Figure 1:**
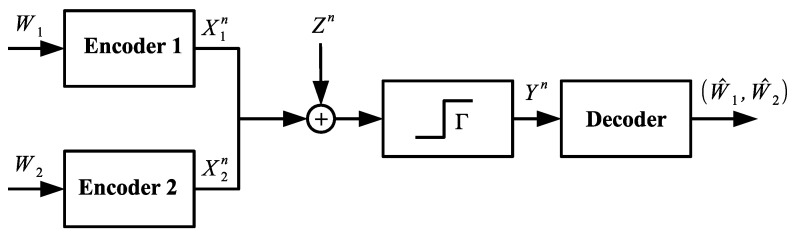
A two-transmitter Gaussian multiple access channel (MAC) with a one-bit analogue-to-digital converter (ADC) front end at the receiver.

We consider a two-transmitter memoryless Gaussian MAC (as shown in Figure 1) with a one-bit quantizer Γ at the receiver front end. Transmitter j=1,2 encodes its message $W_{j}$ into a codeword $X_{j}^{n}$ and transmits it over the shared channel. The signal received by the decoder is given by:

$$Y=\Gamma \left(X_{1,i}+X_{2,i}+Z_{i}\right),\;i\in \left[1:n\right],$$

where ${\left\{Z_{i}\right\}}_{i=1}^{n}$ is an independent and identically distributed (i.i.d.) Gaussian noise process, also independent of the channel inputs $X_{1}^{n}$ and $X_{2}^{n}$ with $Z_{i}∼N\left(0,1\right),i\in \left[1:n\right]$. Γ represents the one-bit ADC operation given by:

$$\Gamma \left(x\right)=\left\{\begin{array}{cc} 1 & x\geq 0\\ 0 & x<0 \end{array}\right..$$

This channel can be modelled by the triplet $\left(X_{1}\times X_{2},p\left(y|x_{1},x_{2}\right),Y\right)$, where $X_{1},X_{2}$ (=R) and Y (={0,1}), respectively, are the alphabets of the inputs and the output. The conditional pmf of the channel output *Y* conditioned on the channel inputs $X_{1}$ and $X_{2}$ (i.e., $p(y|x_{1},x_{2})$) is characterized by:

(1) $$p\left(0|x_{1},x_{2}\right)=1-p\left(1|x_{1},x_{2}\right)=Q\left(x_{1}+x_{2}\right),$$

where $Q\left(x\right)≜\frac{1}{\sqrt{2\pi }}\int_{x}^{+\infty }e^{-\frac{t^{2}}{2}}dt$.

We consider a two-transmitter stationary and memoryless MAC model $\left(X_{1}\times X_{2},p\left(y|x_{1},x_{2}\right),Y\right)$, where $X_{1}=X_{2}=R$, Y={0,1}, $p(y|x_{1},x_{2})$ is given in (1).

A $\left(2^{nR_{1}},2^{nR_{2}},n\right)$ code for this channel consists of (as in [10]):

- two message sets $\left[1:2^{nR_{1}}\right]$ and $\left[1:2^{nR_{2}}\right]$,
- two encoders, where encoder j=1,2 assigns a codeword $x_{j}^{n}\left(w_{j}\right)$ to each message $w_{j}\in \left[1:2^{nR_{j}}\right]$, and
- a decoder that assigns estimates $\left({\hat{w}}_{1},{\hat{w}}_{2}\right)\in \left[1:2^{nR_{1}}\right]\times \left[1:2^{nR_{2}}\right]$ or an error message to each received sequence $y^{n}$.

The stationary property means that the channel does not change over time, while the memoryless property indicates that $p\left(y_{i}|x_{1}^{i},x_{2}^{i},y^{i-1},w_{1},w_{2}\right)=p\left(y_{i}|x_{1,i},x_{2,i}\right)$ for any message pair $\left(w_{1},w_{2}\right)$.

We assume that the message pair $\left(W_{1},W_{2}\right)$ is uniformly distributed over $\left[1:2^{nR_{1}}\right]\times \left[1:2^{nR_{2}}\right]$. The average probability of error is defined as:

(2) $$P_{e}^{\left(n\right)}≜Pr\left\{\left({\hat{W}}_{1},{\hat{W}}_{2}\right)\neq \left(W_{1},W_{2}\right)\right\}.$$

Average power constraints are imposed on the channel inputs as:

(3) $$\frac{1}{n}\sum_{i=1}^{n}x_{j,i}^{2}\left(w_{j}\right)\leq P_{j}\;,\;\forall w_{j}\in \left[1:2^{nR_{j}}\right],\;j\in \left\{1,2\right\},$$

where $x_{j,i}\left(w_{j}\right)$ denotes the *i*-th element of the codeword $x_{j}^{n}\left(w_{j}\right)$.

A rate pair $\left(R_{1},R_{2}\right)$ is said to be achievable for this channel if there exists a sequence of $\left(2^{nR_{1}},2^{nR_{2}},n\right)$ codes satisfying the average power constraints (3), such that $\lim_{n\to \infty }P_{e}^{\left(n\right)}=0$. The capacity region $C(P_{1},P_{2})$ of this channel is the closure of the set of achievable rate pairs $\left(R_{1},R_{2}\right)$.

## 3. Main Results

**Proposition** **1.** *The capacity region $C(P_{1},P_{2})$ of a two-transmitter stationary and memoryless MAC with average power constraints $P_{1}$ and $P_{2}$ is the set of non-negative rate pairs $\left(R_{1},R_{2}\right)$ that satisfy:*

(4) $$\begin{array}{cc} R_{1} & \leq I(X_{1};Y|X_{2},U),\\ R_{2} & \leq I(X_{2};Y|X_{1},U),\\ R_{1}+R_{2} & \leq I(X_{1},X_{2};Y|U), \end{array}$$

*for some $F_{U}\left(u\right)F_{X_{1}|U}\left(x_{1}|u\right)F_{X_{2}|U}\left(x_{2}|u\right)$, such that $E\left[X_{j}^{2}\right]\leq P_{j},\;j=1,2.$ Furthermore, it is sufficient to consider |U|≤5.*

**Proof** **of** **Proposition** **1.** The proof is provided in Appendix A. ☐

The main result of this paper is provided in the following theorem. It bounds the cardinality of the support set of the capacity-achieving distributions.

**Theorem** **1.** *Let J be an arbitrary point on the boundary of the capacity region $C(P_{1},P_{2})$ of the memoryless MAC with a one-bit ADC front end (as shown in Figure 1). J is achieved by a distribution in the form of $F_{U}^{J}\left(u\right)F_{X_{1}|U}^{J}\left(x_{1}|u\right)F_{X_{2}|U}^{J}\left(x_{2}|u\right)$. Furthermore, let $l_{J}$ be the slope of the line tangent to the capacity region at this point. For any u∈U, the conditional input distributions $F_{X_{1}|U}^{J}\left(x_{1}|u\right)$ and $F_{X_{2}|U}^{J}\left(x_{2}|u\right)$ have at most $n_{1}$ and $n_{2}$ points of increase (a point Z is said to be a point of increase of a distribution if for any open set* Ω *containing Z, we have Pr{Ω}>0), respectively, where:*

(5) $$\left(n_{1},n_{2}\right)=\left\{\begin{array}{cc} \left(3,5\right) & l_{J}<-1\\ \left(3,3\right) & l_{J}=-1\\ \left(5,3\right) & l_{J}>-1 \end{array}\right..$$

*Furthermore, this result remains unchanged if the one-bit ADC has a non-zero threshold.*

**Proof** **of** **Theorem** **1.** The proof is provided in Section 4. ☐

Proposition 1 and Theorem 1 establish upper bounds on the number of mass points of the distributions that achieve a boundary point. The significance of this result is that once it is known that the optimal inputs are discrete with at most a certain number of mass points, the capacity region along with the optimal distributions can be obtained via computer programs.

## 4. Proof of Theorem 1

In order to show that the boundary points of the capacity region are achieved, it is sufficient to show that the capacity region is a closed set, i.e., it includes all of its limit points.

Let U be a set with |U|≤5 and Ω be defined as:

(6) $$\Omega ≜\left\{F_{U,X_{1},X_{2}}|\;U\in U,\;X_{1}-U-X_{2},\;E\left[X_{j}^{2}\right]\leq P_{j},\;j=1,2\right\},$$

which is the set of all CDFs on the triplet $\left(U,X_{1},X_{2}\right)$, where *U* is drawn from U, and the Markov chain $X_{1}-U-X_{2}$ and the corresponding average power constraints hold.

In Appendix B, it is proven that Ω is a compact set. Since a continuous mapping preserves compactness, the capacity region is compact. Since the capacity region is a subset of $R^{2}$, it is closed and bounded (note that a subset of $R^{k}$ is compact if and only if it is closed and bounded [11]). Therefore, any point *P* on the boundary of the capacity region is achieved by a distribution denoted by $F_{U}^{J}\left(u\right)F_{X_{1}|U}^{J}\left(x_{1}|u\right)F_{X_{2}|U}^{J}\left(x_{2}|u\right)$.

Since the capacity region is a convex space, it can be characterized by its supporting hyperplanes. In other words, any point on the boundary of the capacity region, denoted by $\left(R_{1}^{b},R_{2}^{b}\right)$, can be written as:

$$\left(R_{1}^{b},R_{2}^{b}\right)=\arg\max_{\left(R_{1},R_{2}\right)\in C\left(P_{1},P_{2}\right)}R_{1}+\lambda R_{2},$$

for some λ∈(0,∞). Here, we have excluded the cases λ=0 and λ=∞, where the channel is not a two-transmitter MAC any longer, and boils down to a point-to-point channel, whose capacity is already known.

Any rate pair $\left(R_{1},R_{2}\right)\in C\left(P_{1},P_{2}\right)$ must lie within a pentagon defined by (4) for some $F_{U}F_{X_{1}|U}F_{X_{2}|U}$ that satisfies the power constraints. Therefore, due to the structure of the pentagon, the problem of finding the boundary points is equivalent to the following maximization problem.

(7) $$\max_{\left(R_{1},R_{2}\right)\in C\left(P_{1},P_{2}\right)}R_{1}+\lambda R_{2}=\left\{\begin{array}{cc} \max I\left(X_{1};Y|X_{2},U\right)+\lambda I\left(X_{2};Y|U\right) & 0<\lambda \leq 1\\ \max I\left(X_{2};Y|X_{1},U\right)+\lambda I\left(X_{1};Y|U\right) & \lambda >1 \end{array}\right.,$$

where on the right-hand side (RHS) of (7), the maximizations are over all $F_{U}F_{X_{1}|U}F_{X_{2}|U}$ that satisfy the power constraints. It is obvious that when λ=1, the two lines in (7) are the same, which results in the sum capacity.

For any product of distributions $F_{X_{1}}F_{X_{2}}$ and the channel in (1), let $I_{\lambda }$ be defined as:

(8) $$I_{\lambda }\left(F_{X_{1}}F_{X_{2}}\right)≜\left\{\begin{array}{cc} I\left(X_{1};Y|X_{2}\right)+\lambda I\left(X_{2};Y\right) & 0<\lambda \leq 1\\ I\left(X_{2};Y|X_{1}\right)+\lambda I\left(X_{1};Y\right) & \lambda >1 \end{array}\right..$$

With this definition, (7) can be rewritten as:

$$\max_{\left(R_{1},R_{2}\right)\in C\left(P_{1},P_{2}\right)}R_{1}+\lambda R_{2}=\max\sum_{i=1}^{5}p_{U}\left(u_{i}\right)I_{\lambda }\left(F_{X_{1}|U}\left(x_{1}|u_{i}\right)F_{X_{2}|U}\left(x_{2}|u_{i}\right)\right),$$

where the second maximization is over distributions of the form $p_{U}\left(u\right)F_{X_{1}|U}\left(x_{1}|u\right)F_{X_{2}|U}\left(x_{2}|u\right)$, such that:

$$\sum_{i=1}^{5}p_{U}\left(u_{i}\right)E\left[X_{j}^{2}|U=u_{i}\right]\leq P_{j},\;j=1,2.$$

**Proposition** **2.** *For a given $F_{X_{1}}$ and any λ>0, $I_{\lambda }\left(F_{X_{1}}F_{X_{2}}\right)$ is a concave, continuous and weakly differentiable function of $F_{X_{2}}$. In the statement of this proposition, $F_{X_{1}}$ and $F_{X_{2}}$ could be interchanged.*

**Proof** **of** **Proposition** **2.** The proof is provided in Appendix C. ☐

**Proposition** **3.** *Let $P_{1}^{\prime },P_{2}^{\prime }$ be two arbitrary non-negative real numbers. For the following problem:*

(9) $$\max_{\begin{array}{c} F_{X_{1}}F_{X_{2}}:\\ E\left[X_{j}^{2}\right]\leq P_{j}^{\prime },\;j=1,2 \end{array}}I_{\lambda }\left(F_{X_{1}}F_{X_{2}}\right),$$

the optimal inputs $F_{X_{1}}^{*}$ and $F_{X_{2}}^{*}$, which are not unique in general, have the following properties,

*(i)*  The support sets of $F_{X_{1}}^{*}$ and $F_{X_{2}}^{*}$ are bounded subsets of R.
*(ii)*  $F_{X_{1}}^{*}$ and $F_{X_{2}}^{*}$ are discrete distributions that have at most $n_{1}$ and $n_{2}$ points of increase, respectively, where:
  $$\left(n_{1},n_{2}\right)=\left\{\begin{array}{cc} \left(5,3\right) & 0<\lambda <1\\ \left(3,3\right) & \lambda =1\\ \left(3,5\right) & \lambda >1 \end{array}\right..$$

**Proof** **of** **Proposition** **3.** We start with the proof of the first claim. Assume that 0<λ≤1, and $F_{X_{2}}$ is given. Consider the following optimization problem:

(10) $$I_{F_{X_{2}}}^{*}≜\underset{\begin{array}{c} F_{X_{1}}:\\ E\left[X_{1}^{2}\right]\leq P_{1}^{\prime } \end{array}}{\sup}I_{\lambda }\left(F_{X_{1}}F_{X_{2}}\right).$$

Note that $I_{F_{X_{2}}}^{*}<+\infty$, since for any λ>0, from (8),

$$I_{\lambda }\leq \left(\lambda +1\right)H\left(Y\right)\leq \left(1+\lambda \right)<+\infty .$$

From Proposition 2, $I_{\lambda }$ is a continuous, concave function of $F_{X_{1}}$. Furthermore, the set of all CDFs with bounded second moment (here, $P_{1}^{\prime }$) is convex and compact. The compactness follows from Appendix I in [12], where the only difference is in using Chebyshev’s inequality instead of Markov’s inequality. Therefore, the supremum in (10) is achieved by a distribution $F_{X_{1}}^{*}$. Since for any $F_{X_{1}}\left(x\right)=s\left(x-x_{0}\right)$ with $|x_{0}{|}^{2}<P_{1}^{\prime }$, we have $E\left[X_{1}^{2}\right]<P_{1}^{\prime }$, the Lagrangian theorem and the Karush–Kuhn–Tucker conditions state that there exists a $\theta_{1}\geq 0$ such that:

(11) $$\begin{array}{cc} I_{F_{X_{2}}}^{*} & =\underset{F_{X_{1}}}{\sup}\left\{I_{\lambda }\left(F_{X_{1}}F_{X_{2}}\right)-\theta_{1}\left(\int x^{2}dF_{X_{1}}\left(x\right)-P_{1}^{\prime }\right)\right\}. \end{array}$$

Furthermore, the supremum in (11) is achieved by $F_{X_{1}}^{*}$, and:

(12) $$\theta_{1}\left(\int x^{2}dF_{X_{1}}^{*}\left(x\right)-P_{1}^{\prime }\right)=0.$$

**Lemma** **1.** *The Lagrangian multiplier $\theta_{1}$ is non-zero. From (12), this is equivalent to having $E\left[X_{1}^{2}\right]=P_{1}^{\prime }$, i.e., the first user transmits with its maximum allowable power (note that this is for λ≤1, as used in Appendix D).*

**Proof** **of** **Lemma** **1.** In what follows, we prove that a zero Lagrangian multiplier is not possible. Having a zero Lagrangian multiplier means the power constraint is inactive. In other words, if $\theta_{1}=0$, (10) and (11) imply that:

(13) $$\underset{\begin{array}{c} F_{X_{1}}\\ E\left[X_{1}^{2}\right]\leq P_{1}^{\prime } \end{array}}{\sup}I_{\lambda }\left(F_{X_{1}}F_{X_{2}}\right)=\underset{F_{X_{1}}}{\sup}I_{\lambda }\left(F_{X_{1}}F_{X_{2}}\right).$$

We prove that (13) does not hold by showing that its left-hand side (LHS) is strictly less than one, while its RHS equals one. The details are provided in Appendix D. ☐

$I_{\lambda }\left(F_{X_{1}}F_{X_{2}}\right)$ (0<λ≤1) can be written as:

(14) $$\begin{array}{cc} I_{\lambda }\left(F_{X_{1}}F_{X_{2}}\right) & =\int_{-\infty }^{+\infty }\int_{-\infty }^{+\infty }\sum_{y=0}^{1}p\left(y|x_{1},x_{2}\right)\log\frac{p(y|x_{1},x_{2})}{{\left[p\left(y;F_{X_{1}}F_{X_{2}}\right)\right]}^{\lambda }{\left[p\left(y;F_{X_{1}}|x_{2}\right)\right]}^{1-\lambda }}dF_{X_{1}}\left(x_{1}\right)dF_{X_{2}}\left(x_{2}\right)\\ & =\int_{-\infty }^{+\infty }{\tilde{i}}_{\lambda }\left(x_{1};F_{X_{1}}|F_{X_{2}}\right)dF_{X_{1}}\left(x_{1}\right) \end{array}$$

(15) $$\begin{array}{cc} & =\int_{-\infty }^{+\infty }i_{\lambda }\left(x_{2};F_{X_{2}}|F_{X_{1}}\right)dF_{X_{2}}\left(x_{2}\right), \end{array}$$

where we have defined:

(16) $${\tilde{i}}_{\lambda }\left(x_{1};F_{X_{1}}|F_{X_{2}}\right)\;≜\;\;\int_{-\infty }^{+\infty }\;\;\left(D\left(p\left(y|x_{1},x_{2}\right)\left|\right|p\left(y;F_{X_{1}}F_{X_{2}}\right)\right)+\left(1-\lambda \right)\sum_{y=0}^{1}p\left(y|x_{1},x_{2}\right)\log\frac{p(y;F_{X_{1}}F_{X_{2}})}{p(y;F_{X_{1}}|x_{2})}\right)\;\;dF_{X_{2}}\left(x_{2}\right),$$

and:

(17) $$i_{\lambda }\left(x_{2};F_{X_{2}}|F_{X_{1}}\right)≜\int_{-\infty }^{+\infty }D\left(p\left(y|x_{1},x_{2}\right)\left|\right|p\left(y;F_{X_{1}}F_{X_{2}}\right)\right)dF_{X_{1}}\left(x_{1}\right)-\left(1-\lambda \right)D\left(p\left(y;F_{X_{1}}|x_{2}\right)\left|\right|p\left(y;F_{X_{1}}F_{X_{2}}\right)\right).$$

$p(y;F_{X_{1}}F_{X_{2}})$ is nothing but the pmf of *Y* with the emphasis that it has been induced by $F_{X_{1}}$ and $F_{X_{2}}$. Likewise, $p(y;F_{X_{1}}|x_{2})$ is the conditional pmf $p(y|x_{2})$ when $X_{1}$ is drawn according to $F_{X_{1}}$. From (14), ${\tilde{i}}_{\lambda }\left(x_{1};F_{X_{1}}|F_{X_{2}}\right)$ can be considered as the density of $I_{\lambda }$ over $F_{X_{1}}$ when $F_{X_{2}}$ is given. $i_{\lambda }\left(x_{2};F_{X_{2}}|F_{X_{1}}\right)$ can be interpreted in a similar way.

Note that (11) is an unconstrained optimization problem over the set of all CDFs. Since $\int x^{2}dF_{X_{1}}\left(x\right)$ is linear and weakly differentiable in $F_{X_{1}}$, the objective function in (11) is concave and weakly differentiable. Hence, a necessary condition for the optimality of $F_{X_{1}}^{*}$ is:

(18) $$\int \left\{{\tilde{i}}_{\lambda }\left(x_{1};F_{X_{1}}^{*}|F_{X_{2}}\right)+\theta_{1}\left(P_{1}^{\prime }-x_{1}^{2}\right)\right\}dF_{X_{1}}\left(x_{1}\right)\leq I_{F_{X_{2}}}^{*},\;\;\forall F_{X_{1}}.$$

Furthermore, (18) can be verified to be equivalent to:

(19) $$\begin{array}{cc} {\tilde{i}}_{\lambda }\left(x_{1};F_{X_{1}}^{*}|F_{X_{2}}\right)+\theta_{1}\left(P_{1}^{\prime }-x_{1}^{2}\right) & \leq I_{F_{X_{2}}}^{*},\;\;\forall x_{1}\in R, \end{array}$$

(20) $$\begin{array}{cc} {\tilde{i}}_{\lambda }\left(x_{1};F_{X_{1}}^{*}|F_{X_{2}}\right)+\theta_{1}\left(P_{1}^{\prime }-x_{1}^{2}\right) & =I_{F_{X_{2}}}^{*},\;\;\mathrm{if}\;x_{1}\;\mathrm{is}\;a\;\mathrm{point}\;\mathrm{of}\;\mathrm{increase}\;\mathrm{of}\;F_{X_{1}}^{*}. \end{array}$$

The justifications of (18)–(20) are provided in Appendix E.

In what follows, we prove that in order to satisfy (20), $F_{X_{1}}^{*}$ must have a bounded support by showing that the LHS of (20) goes to −∞ with $x_{1}$. The following lemma is useful in the sequel for taking the limit processes inside the integrals.

**Lemma** **2.** *Let $X_{1}$ and $X_{2}$ be two independent random variables satisfying $E\left[X_{1}^{2}\right]\leq P_{1}^{\prime }$ and $E\left[X_{2}^{2}\right]\leq P_{2}^{\prime }$, respectively ($P_{1}^{\prime },P_{2}^{\prime }\in \left[0,+\infty \right)$). Considering the conditional pmf in (1), the following inequalities hold.*

(21) $$\begin{array}{cc} \left|D\left(p\left(y|x_{1},x_{2}\right)\left|\right|p\left(y;F_{X_{1}}F_{X_{2}}\right)\right)\right| & \leq 1-2\log Q(\sqrt{P_{1}^{\prime }}+\sqrt{P_{2}^{\prime }}) \end{array}$$

(22) $$\begin{array}{cc} p(y;F_{X_{1}}|x_{2}) & \geq Q\left(\sqrt{P_{1}^{\prime }}+\left|x_{2}\right|\right) \end{array}$$

(23) $$\begin{array}{cc} \left|\sum_{y=0}^{1}p\left(y|x_{1},x_{2}\right)\log\frac{p(y;F_{X_{1}}F_{X_{2}})}{p(y;F_{X_{1}}|x_{2})}\right| & \leq -2\log Q\left(\sqrt{P_{1}^{\prime }}+\sqrt{P_{2}^{\prime }}\right)-2\log Q\left(\sqrt{P_{1}^{\prime }}+\left|x_{2}\right|\right) \end{array}$$

**Proof** **of** **Lemma** **2.** The proof is provided in Appendix F. ☐

Note that

(24) $$\begin{array}{cc} \lim_{x_{1}\to +\infty }\int_{-\infty }^{+\infty }D\left(p\left(y|x_{1},x_{2}\right)\left|\right|p\left(y;F_{X_{1}}^{*}F_{X_{2}}\right)\right)dF_{X_{2}}\left(x_{2}\right) & =\int_{-\infty }^{+\infty }\lim_{x_{1}\to +\infty }D\left(p\left(y|x_{1},x_{2}\right)\left|\right|p\left(y;F_{X_{1}}^{*}F_{X_{2}}\right)\right)dF_{X_{2}}\left(x_{2}\right) \end{array}$$

(25) $$\begin{array}{cc} \; & =-\log p_{Y}\left(1;F_{X_{1}}^{*}F_{X_{2}}\right) \end{array}$$

(26) $$\begin{array}{cc} \; & \leq -\log Q(\sqrt{P_{1}^{\prime }}+\sqrt{P_{2}^{\prime }}), \end{array}$$

where (24) is due to the Lebesgue dominated convergence theorem [11] and (21), which permit the interchange of the limit and the integral; (25) is due to the following:

$$\begin{array}{cc} \lim_{x_{1}\to +\infty }D\left(p\left(y|x_{1},x_{2}\right)\left|\right|p\left(y;F_{X_{1}}^{*}F_{X_{2}}\right)\right) & =\lim_{x_{1}\to +\infty }\sum_{y=0}^{1}p\left(y|x_{1},x_{2}\right)\log\frac{p(y|x_{1},x_{2})}{p(y;F_{X_{1}}^{*}F_{X_{2}})}\\ & =-\log p_{Y}\left(1;F_{X_{1}}^{*}F_{X_{2}}\right), \end{array}$$

since $p\left(0|x_{1},x_{2}\right)=Q\left(x_{1}+x_{2}\right)$ goes to zero when $x_{1}\to +\infty$ and $p_{Y}\left(y;F_{X_{1}}^{*}F_{X_{2}}\right)\;\left(y=0,1\right)$ is bounded away from zero by (A34) ; (26) is obtained from (A34) in Appendix F. Furthermore,

(27) $$\begin{array}{cc} \lim_{x_{1}\to +\infty }\int_{-\infty }^{+\infty }\sum_{y=0}^{1}p\left(y|x_{1},x_{2}\right)\log\frac{p(y;F_{X_{1}}^{*}F_{X_{2}})}{p(y;F_{X_{1}}^{*}|x_{2})}dF_{X_{2}}\left(x_{2}\right) & =\;\int_{-\infty }^{+\infty }\;\;\;\;\lim_{x_{1}\to +\infty }\sum_{y=0}^{1}p\left(y|x_{1},x_{2}\right)\log\frac{p(y;F_{X_{1}}^{*}F_{X_{2}})}{p(y;F_{X_{1}}^{*}|x_{2})}dF_{X_{2}}\left(x_{2}\right) \end{array}$$

(28) $$\begin{array}{cc} \; & \;\;\;=\log p_{Y}\left(1;F_{X_{1}}^{*}F_{X_{2}}\right)-\int_{-\infty }^{+\infty }\log p\left(1;F_{X_{1}}^{*}|x_{2}\right)dF_{X_{2}}\left(x_{2}\right)\\ \; & \;\;\;<-\log Q\left(\sqrt{P_{1}^{\prime }}+\sqrt{P_{2}^{\prime }}\right), \end{array}$$

where (27) is due to the Lebesgue dominated convergence theorem along with (23) and (A39) in Appendix F; (28) is from (22) and the convexity of $\log Q(\alpha +\sqrt{t})$ in *t* when α≥0 (see Appendix G).

Therefore, from (26) and (28),

(29) $$\begin{array}{c} \lim_{x_{1}\to +\infty }{\tilde{i}}_{\lambda }\left(x_{1};F_{X_{1}}^{*}|F_{X_{2}}\right)\leq -\left(2-\lambda \right)\log Q\left(\sqrt{P_{1}^{\prime }}+\sqrt{P_{2}^{\prime }}\right)<+\infty . \end{array}$$

Using a similar approach, we can also obtain:

(30) $$\begin{array}{c} \lim_{x_{1}\to -\infty }{\tilde{i}}_{\lambda }\left(x_{1};F_{X_{1}}^{*}|F_{X_{2}}\right)\leq -\left(2-\lambda \right)\log Q\left(\sqrt{P_{1}^{\prime }}+\sqrt{P_{2}^{\prime }}\right)<+\infty . \end{array}$$

From (29) and (30) and the fact that $\theta_{1}>0$ (see Lemma 1), the LHS of (19) goes to −∞ when $|x_{1}|\to +\infty$. Since any point of increase of $F_{X_{1}}^{*}$ must satisfy (19) with equality and $I_{F_{X_{2}}}^{*}\geq 0$, it is proven that $F_{X_{1}}^{*}$ has a bounded support. Hence, from now on, we assume $X_{1}\in \left[-A_{1},A_{2}\right]$ for some $A_{1},A_{2}\in R$ (note that $A_{1}$ and $A_{2}$ are determined by the choice of $F_{X_{2}}$).

Similarly, for a given $F_{X_{1}}$, the optimization problem:

$$I_{F_{X_{1}}}^{*}=\underset{\begin{array}{c} F_{X_{2}}:\\ E\left[X_{2}^{2}\right]\leq P_{2}^{\prime } \end{array}}{\sup}I_{\lambda }\left(F_{X_{1}}F_{X_{2}}\right),$$

boils down to the following necessary condition:

(31) $$\begin{array}{cc} i_{\lambda }\left(x_{2};F_{X_{2}}^{*}|F_{X_{1}}\right)+\theta_{2}\left(P_{2}^{\prime }-x_{2}^{2}\right) & \leq I_{F_{X_{1}}}^{*},\;\;\forall x_{2}\in R, \end{array}$$

(32) $$\begin{array}{cc} i_{\lambda }\left(x_{2};F_{X_{2}}^{*}|F_{X_{1}}\right)+\theta_{2}\left(P_{2}^{\prime }-x_{2}^{2}\right) & =I_{F_{X_{1}}}^{*},\;\;\mathrm{if}\;x_{2}\;\mathrm{is}\;a\;\mathrm{point}\;\mathrm{of}\;\mathrm{increase}\;\mathrm{of}\;F_{X_{2}}^{*}, \end{array}$$

for the optimality of $F_{X_{2}}^{*}$. However, there are two main differences between (32) and (20). First is the difference between $i_{\lambda }$ and ${\tilde{i}}_{\lambda }$. Second is the fact that we do not claim $\theta_{2}$ to be nonzero, since the approach used in Lemma 1 cannot be readily applied to $\theta_{2}$. Nonetheless, the boundedness of the support of $F_{X_{2}}^{*}$ can be proven by inspecting the behaviour of the LHS of (32) when $|x_{2}|\to +\infty$.

In what follows, i.e., from (33)–(38), we prove that the support of $F_{X_{2}}^{*}$ is bounded by showing that (32) does not hold when $|x_{2}|$ is above a certain threshold. The first term on the LHS of (32) is $i_{\lambda }\left(x_{2};F_{X_{2}}^{*}|F_{X_{1}}\right)$. From (17) and (21), it can be easily verified that:

(33) $$\begin{array}{cc} \lim_{x_{2}\to +\infty }i_{\lambda }\left(x_{2};F_{X_{2}}^{*}|F_{X_{1}}\right) & =-\lambda \log p_{Y}\left(1;F_{X_{1}}F_{X_{2}}^{*}\right)\leq -\lambda \log Q\left(\sqrt{P_{1}^{\prime }}+\sqrt{P_{2}^{\prime }}\right),\\ \lim_{x_{2}\to -\infty }i_{\lambda }\left(x_{2};F_{X_{2}}^{*}|F_{X_{1}}\right) & =-\lambda \log p_{Y}\left(0;F_{X_{1}}F_{X_{2}}^{*}\right)\leq -\lambda \log Q\left(\sqrt{P_{1}^{\prime }}+\sqrt{P_{2}^{\prime }}\right) \end{array}$$

From (33), if $\theta_{2}>0$, the LHS of (32) goes to −∞ with $|x_{2}|$, which proves that $X_{2}^{*}$ is bounded.

For the possible case of $\theta_{2}=0$, in order to show that (32) does not hold when $|x_{2}|$ is above a certain threshold, we rely on the boundedness of $X_{1}$, i.e., $X_{1}\in \left[-A_{1},A_{2}\right]$. Then, we prove that $i_{\lambda }$ approaches its limit in (33) from below. In other words, there is a real number *K* such that $i_{\lambda }\left(x_{2};F_{X_{2}}^{*}|F_{X_{1}}\right)<-\lambda \log p_{Y}\left(1;F_{X_{1}}F_{X_{2}}^{*}\right)$ when $x_{2}>K$, and $i_{\lambda }\left(x_{2};F_{X_{2}}^{*}|F_{X_{1}}\right)<-\lambda \log p_{Y}\left(0;F_{X_{1}}F_{X_{2}}^{*}\right)$ when $x_{2}<-K$. This establishes the boundedness of $X_{2}^{*}$. In what follows, we only show the former, i.e., when $x_{2}\to +\infty$. The latter, i.e., $x_{2}\to -\infty$, follows similarly, and it is omitted for the sake of brevity.

By rewriting $i_{\lambda }$, we have:

(34) $$\begin{array}{cc} i_{\lambda }\left(x_{2};F_{X_{2}}^{*}|F_{X_{1}}\right) & =-\lambda p\left(1;F_{X_{1}}|x_{2}\right)\log p_{Y}\left(1;F_{X_{1}}F_{X_{2}}^{*}\right)\\ & \;-\int_{-A_{1}}^{A_{2}}H_{b}\left(Q\left(x_{1}+x_{2}\right)\right)dF_{X_{1}}\left(x_{1}\right)+\left(1-\lambda \right)\;\;\;\;\;\;\;\underset{H_{b}\left(\int Q\left(x_{1}+x_{2}\right)dF_{X_{1}}\left(x_{1}\right)\right)}{\underset{︸}{H(Y|X_{2}=x_{2})}}\\ & -\lambda \;\;\;\;\;\underset{\int Q\left(x_{1}+x_{2}\right)dF_{X_{1}}\left(x_{1}\right)}{\underset{︸}{p(0;F_{X_{1}}|x_{2})}}\;\;\;\log p_{Y}\left(0;F_{X_{1}}F_{X_{2}}^{*}\right). \end{array}$$

It is obvious that the first term on the RHS of (34) approaches $-\lambda \log p_{Y}\left(1;F_{X_{1}}F_{X_{2}}^{*}\right)$ from below when $x_{2}\to +\infty$, since $p(1;F_{X_{1}}|x_{2})\leq 1$. It is also obvious that the remaining terms go to zero when $x_{2}\to +\infty$. Hence, it is sufficient to show that they approach zero from below, which is proven by using the following lemma.

**Lemma** **3.** *Let $X_{1}$ be distributed on $\left[-A_{1},A_{2}\right]$ according to $F_{X_{1}}\left(x_{1}\right)$. We have:*

(35) $$\begin{array}{c} \lim_{x_{2}\to +\infty }\frac{\int_{-A_{1}}^{A_{2}}H_{b}\left(Q\left(x_{1}+x_{2}\right)\right)dF_{X_{1}}\left(x_{1}\right)}{H_{b}\left(\int_{-A_{1}}^{A_{2}}Q\left(x_{1}+x_{2}\right)dF_{X_{1}}\left(x_{1}\right)\right)}=1. \end{array}$$

**Proof** **of** **Lemma** **3.** The proof is provided in Appendix H. ☐

From (35), we can write:

(36) $$\int_{-A_{1}}^{A_{2}}H_{b}\left(Q\left(x_{1}+x_{2}\right)\right)dF_{X_{1}}\left(x_{1}\right)=\gamma \left(x_{2}\right)H_{b}\left(\int_{-A_{1}}^{A_{2}}Q\left(x_{1}+x_{2}\right)dF_{X_{1}}\left(x_{1}\right)\right),$$

where $\gamma (x_{2})\leq 1$ (due to the concavity of $H_{b}\left(\cdot \right)$), and $\gamma (x_{2})\to 1$ when $x_{2}\to +\infty$ (due to (35)). Furthermore, from the fact that $\lim_{x\to 0}\frac{H_{b}\left(x\right)}{cx}=+\infty \;\left(c>0\right)$, we have:

(37) $$H_{b}\left(\int_{-A_{1}}^{A_{2}}Q\left(x_{1}+x_{2}\right)dF_{X_{1}}\left(x_{1}\right)\right)=-\eta \left(x_{2}\right)\log p_{Y}\left(0;F_{X_{1}}F_{X_{2}}^{*}\right)\int_{-A_{1}}^{A_{2}}Q\left(x_{1}+x_{2}\right)dF_{X_{1}}\left(x_{1}\right),$$

where $\eta (x_{2})>0$ and $\eta (x_{2})\to +\infty$ when $x_{2}\to +\infty$. From (36)–(37), the second and the third line of (34) become:

(38) $$\begin{array}{c} \left(1-\gamma \left(x_{2}\right)+\frac{\lambda }{\eta (x_{2})}-\lambda \right)\underset{\geq 0}{\underset{︸}{\left(-\eta \left(x_{2}\right)\log p_{Y}\left(0;F_{X_{1}}F_{X_{2}}^{*}\right)\int_{-A_{1}}^{A_{2}}Q\left(x_{1}+x_{2}\right)dF_{X_{1}}\left(x_{1}\right)\right)}}. \end{array}$$

Since $\gamma (x_{2})\to 1$ and $\eta (x_{2})\to +\infty$ as $x_{2}\to +\infty$, there exists a real number *K* such that $1-\gamma \left(x_{2}\right)+\frac{\lambda }{\eta (x_{2})}-\lambda <0$ when $x_{2}>K$. Therefore, the second and the third line of (34) approach zero from below, which proves that the support of $X_{2}^{*}$ is bounded away from +∞. As mentioned before, a similar argument holds when $x_{2}\to -\infty$. This proves that $X_{2}^{*}$ has a bounded support.

**Remark** **1.** We remark here that the order of showing the boundedness of the supports is important. First, for a given $F_{X_{2}}$ (not necessarily bounded), it is proven that $F_{X_{1}}^{*}$ is bounded. Then, for a given bounded $F_{X_{1}}$, it is shown that $F_{X_{2}}^{*}$ is also bounded. Hence, the boundedness of the supports of the optimal input distributions is proven by contradiction. The order is reversed when λ>1, and it follows the same steps as in the case of λ≤1. Therefore, it is omitted.

We next prove the second claim in Proposition 3. We assume that 0<λ<1, and a bounded $F_{X_{1}}$ is given. We already know that for a given bounded $F_{X_{1}}$, $F_{X_{2}}^{*}$ has a bounded support denoted by $\left[-B_{1},B_{2}\right]$. Therefore,

(39) $$\begin{array}{c} I_{F_{X_{1}}}^{*}=\underset{\begin{array}{c} F_{X_{2}}:\\ E\left[X_{2}^{2}\right]\leq P_{2}^{\prime } \end{array}}{\sup}I_{\lambda }\left(F_{X_{1}}F_{X_{2}}\right)\\ I_{F_{X_{1}}}^{*}=\underset{\begin{array}{c} F_{X_{2}}\in S_{2}:\\ E\left[X_{2}^{2}\right]\leq P_{2}^{\prime } \end{array}}{\sup}I_{\lambda }\left(F_{X_{1}}F_{X_{2}}\right), \end{array}$$

where $S_{2}$ denotes the set of all probability distributions on the Borel sets of $\left[-B_{1},B_{2}\right]$. Let $p_{0}^{*}=p_{Y}\left(0;F_{X_{1}}F_{X_{2}}^{*}\right)$ denote the probability of the event Y=0, induced by $F_{X_{2}}^{*}$ and the given $F_{X_{1}}$. Furthermore, let $P_{2}^{*}$ denote the second moment of $X_{2}$ under $F_{X_{2}}^{*}$. The set:

(40) $$F_{2}=\left\{F_{X_{2}}\in S_{2}|\int_{-B_{1}}^{B_{2}}p\left(0|x_{2}\right)dF_{X_{2}}\left(x_{2}\right)=p_{0}^{*},\;\int_{-B_{1}}^{B_{2}}x_{2}^{2}dF_{X_{2}}\left(x_{2}\right)=P_{2}^{*}\right\}$$

is the intersection of $S_{2}$ with two hyperplanes (note that $S_{2}$ is convex and compact). We can write:

(41) $$I_{F_{X_{1}}}^{*}=\underset{F_{X_{2}}\in F_{2}}{\sup}I_{\lambda }\left(F_{X_{1}}F_{X_{2}}\right).$$

Note that having $F_{X_{2}}\in F_{2}$, the objective function in (41) becomes:

(42) $$\begin{array}{c} \underset{\mathrm{constant}}{\underset{︸}{\lambda H(Y)}}+\underset{\mathrm{linear}\mathrm{in}F_{X_{2}}}{\underset{︸}{\left(1-\lambda \right)H\left(Y|X_{2}\right)-H\left(Y|X_{1},X_{2}\right)}}. \end{array}$$

Since the linear part is continuous and $F_{2}$ is compact (The continuity of the linear part follows similarly the continuity arguments in Appendix C. Note that this compactness is due to the closedness of the intersecting hyperplanes in $F_{2}$, since a closed subset of a compact set is compact [11]. The hyperplanes are closed due to the continuity of $x_{2}^{2}$ and $p(0|x_{2})$ (see (A16)).), the objective function in (41) attains its maximum at an extreme point of $F_{2}$, which, by Dubins’ theorem, is a convex combination of at most three extreme points of $S_{2}$. Since the extreme points of $S_{2}$ are the CDFs having only one point of increase in $\left[-B_{1},B_{2}\right]$, we conclude that given any bounded $F_{X_{1}}$, $F_{X_{2}}^{*}$ has at most three mass points.

Now, assume that an arbitrary $F_{X_{2}}$ is given with at most three mass points denoted by ${\left\{x_{2,i}\right\}}_{i=1}^{3}$. It is already known that the support of $F_{X_{1}}^{*}$ is bounded, which is denoted by $\left[-A_{1},A_{2}\right]$. Let $S_{1}$ denote the set of all probability distributions on the Borel sets of $\left[-A_{1},A_{2}\right]$. The set:

(43) $$\begin{array}{c} F_{1}=\left\{F_{X_{1}}\in S_{1}|\int_{-A_{1}}^{A_{2}}p\left(0|x_{1},x_{2,j}\right)dF_{X_{1}}\left(x_{1}\right)=p\left(0;F_{X_{1}}^{*}|x_{2,j}\right),\;j\in \left[1:3\right],\;\int_{-A_{1}}^{A_{2}}x_{1}^{2}dF_{X_{1}}\left(x_{1}\right)=P_{1}^{\prime }\right\}, \end{array}$$

is the intersection of $S_{1}$ with four hyperplanes. Note that here, since we know $\theta_{1}\neq 0$, the optimal input attains its maximum power of $P_{1}^{\prime }$. In a similar way,

(44) $$I_{F_{X_{2}}}^{*}=\underset{F_{X_{1}}\in F_{1}}{\sup}\left\{I_{\lambda }\left(F_{X_{1}}F_{X_{2}}\right)\right\},$$

and having $F_{X_{1}}\in F_{1}$, the objective function in (44) becomes:

(45) $$\begin{array}{cc} I_{\lambda } & =\underset{\mathrm{constant}}{\underset{︸}{\lambda H\left(Y\right)+\left(1-\lambda \right)\sum_{i=1}^{3}p_{X_{2}}\left(x_{2,i}\right)H\left(Y|X_{2}=x_{2,i}\right)}}-\underset{\mathrm{linear}\mathrm{in}F_{X_{1}}}{\underset{︸}{H(Y|X_{1},X_{2})}} \end{array}$$

Therefore, given any $F_{X_{2}}$ with at most three points of increase, $F_{X_{1}}^{*}$ has at most five mass points.

When λ=1, the second term on the RHS of (45) disappears, which means that $F_{1}$ could be replaced by:

$$\left\{F_{X_{1}}\in S_{1}|\int_{-A_{1}}^{A_{2}}p\left(0|x_{1}\right)dF_{X_{1}}\left(x_{1}\right)={\tilde{p}}_{0}^{*},\;\int_{-A_{1}}^{A_{2}}x_{1}^{2}dF_{X_{1}}\left(x_{1}\right)=P_{1}^{\prime }\right\},$$

where ${\tilde{p}}_{0}^{*}=p_{Y}\left(0;F_{X_{1}}^{*}F_{X_{2}}\right)$ is the probability of the event Y=0, which is induced by $F_{X_{1}}^{*}$ and the given $F_{X_{2}}$. Since the number of intersecting hyperplanes has been reduced to two, it is concluded that $F_{X_{1}}^{*}$ has at most three points of increase. ☐

**Remark** **2.** Note that, the order of showing the discreteness of the support sets is also important. First, for a given bounded $F_{X_{1}}$ (not necessarily discrete), it is proven that $F_{X_{2}}^{*}$ is discrete with at most three mass points. Then, for a given discrete $F_{X_{2}}$ with at most three mass points, it is shown that $F_{X_{1}}^{*}$ is also discrete with at most five mass points when λ<1 and at most three mass points when λ=1. When λ>1, the order is reversed, and it follows the same steps as in the case of λ<1. Therefore, it is omitted.

**Remark** **3.** *If $X_{1},X_{2}$ are assumed finite initially, similar results can be obtained by using the iterative optimization in the previous proof and the approach in Chapter 4, Corollary 3 of [13].*

## 5. Sum Rate Analysis

In this section, we propose a lower bound on the sum capacity of a MAC in the presence of a one-bit ADC front end at the receiver, which we conjecture to be tight. The sum capacity is given by:

(46) $$C_{\mathrm{sum}}=\sup\;I\left(X_{1},X_{2};Y|U\right),$$

where the supremum is over $F_{U}F_{X_{1}|U}F_{X_{2}|U}$ (|U|≤5), such that $E\left[X_{j}^{2}\right]\leq P_{j},\;j=1,2$. We obtain a lower bound for the above by considering only those input distributions that are zero-mean per any realization of the auxiliary random variable *U*, i.e., $E[X_{j}|U=u]=0,\forall u\in U,j=1,2$. Let $P_{1}^{\prime }$ and $P_{2}^{\prime }$ be two arbitrary non-negative real numbers. We have:

(47) $$\begin{array}{cc} \underset{\begin{array}{c} F_{X_{1}}F_{X_{2}}:\\ E\left[X_{j}^{2}\right]\leq P_{j}^{\prime }\\ E[X_{j}]=0,\;j=1,2 \end{array}}{\sup}I\left(X_{1},X_{2};Y\right) & \leq \underset{\begin{array}{c} F_{\tilde{X}}:\\ E\left[{\tilde{X}}^{2}\right]\leq P_{1}^{\prime }+P_{2}^{\prime } \end{array}}{\sup}I\left(\tilde{X};Y\right) \end{array}$$

(48) $$\begin{array}{c} =1-H_{b}\left(Q\left(\sqrt{P_{1}^{\prime }+P_{2}^{\prime }}\right)\right) \end{array}$$

where in (47), $\tilde{X}≜X_{1}+X_{2}$, $p_{Y|\tilde{X}}\left(0|\tilde{x}\right)=Q\left(\tilde{x}\right)$; (48) follows from [4] for the point-to-point channel. Therefore, when $E[X_{j}|U=u]=0,\forall u\in U,j=1,2,$ we can write:

(49) $$\begin{array}{cc} I(X_{1},X_{2};Y|U) & =\sum_{i=1}^{5}p_{U}\left(u_{i}\right)I\left(X_{1},X_{2};Y|U=u_{i}\right)\\ & \leq 1-\sum_{i=1}^{5}p_{U}\left(u_{i}\right)H_{b}\left(Q\left(\sqrt{E\left[X_{1}^{2}|U=u_{i}\right]+E\left[X_{2}^{2}|U=u_{i}\right]}\right)\right)\\ & \leq 1-H_{b}\left(Q\left(\sqrt{E\left[X_{1}^{2}\right]+E\left[X_{2}^{2}\right]}\right)\right) \end{array}$$

(50) $$\begin{array}{c} \leq 1-H_{b}\left(Q\left(\sqrt{P_{1}+P_{2}}\right)\right), \end{array}$$

where (49) is due to the fact that $H_{b}\left(Q(\sqrt{x+y})\right)$ is a convex function of (x,y), and (50) follows from $E\left[X_{j}^{2}\right]\leq P_{j},j=1,2$.

The upper bound in (50) can be achieved by time division with power control as follows. Let U={0,1} and $p_{U}\left(0\right)=1-p_{U}\left(1\right)=\frac{P_{1}}{P_{1}+P_{2}}$. Furthermore, let $F_{X_{1}|U}\left(x|1\right)=F_{X_{2}|U}\left(x|0\right)=s\left(x\right)$, where s(·) is the unit step function, and:

$$\begin{array}{c} F_{X_{1}|U}\left(x|0\right)=F_{X_{2}|U}\left(x|1\right)=\frac{1}{2}s\left(x+\sqrt{P_{1}+P_{2}}\right)+\frac{1}{2}s\left(x-\sqrt{P_{1}+P_{2}}\right). \end{array}$$

With this choice of $F_{U}F_{X_{1}|U}F_{X_{2}|U}$, the upper bound in (50) is achieved. Therefore,

(51) $$C_{sum}\geq 1-H_{b}\left(Q\left(\sqrt{P_{1}+P_{2}}\right)\right).$$

A numerical evaluation of (46) is carried out as follows (the codes that are used for the numerical simulations are available at https://www.dropbox.com/sh/ndxkjt6h5a0yktu/AAAmfHkuPxe8rMNV1KzFVRgNa?dl=0). Although $E[X_{j}^{2}]$ is upper bounded by $P_{j}$ (j=1,2), the value of $E[X_{j}^{2}|U=u]$ (∀u∈U) has no upper bound and could be any non-negative real number. However, in our numerical analysis, we further restrict our attention to the case $E\left[X_{j}^{2}|U=u\right]\leq 20P_{j},\forall u\in U,j=1,2.$ Obviously, as this upper bound tends to infinity, the approximation becomes more accurate (This further bounding of the conditional second moments is justified by the fact that the sum capacity is not greater than one, which is due to the one-bit quantization at the receiver. As a result, $I(X_{1},X_{2};Y|U=u)$ increases at most sublinearly with $E[X_{j}^{2}|U=u],j=1,2$, while $p_{U}\left(u\right)$ needs to decrease at least linearly to satisfy the average power constraints. Hence, the product $p_{U}\left(u\right)I\left(X_{1},X_{2};Y|U=u\right)$ decreases with $E[X_{j}^{2}|U=u]$ when $E[X_{j}^{2}|U=u]$ is above a threshold.). Each of the intervals $\left[0,20P_{1}\right]$ and $\left[0,20P_{2}\right]$ are divided into 201 points uniformly, which results in the discrete intervals $\frac{P_{1}}{10}\left[0:200\right]$ and $\frac{P_{2}}{10}\left[0:200\right]$, respectively. Afterwards, for any pair $\left(\alpha ,\beta \right)\in \frac{P_{1}}{10}\left[0:200\right]\times \frac{P_{2}}{10}\left[0:200\right]$, the following is carried out for input distributions with at most three mass points.

(52) $$\max_{\begin{array}{c} F_{X_{1}}F_{X_{2}}:\\ E\left[X_{1}^{2}\right]\leq \alpha ,E\left[X_{2}^{2}\right]\leq \beta \end{array}}I\left(X_{1},X_{2};Y\right)$$

The results are stored in a 201×201 matrix accordingly. In the above optimization, the MATLAB function fmincon is used with three different initial values, and the maximum of these three experiments is chosen. Then, the problem boils down to finding proper gains, i.e., the mass probabilities of *U*, that maximize $I(X_{1},X_{2};Y|U)$ and satisfy the average power constraints $E\left[X_{j}^{2}\right]\leq P_{j}$. This is done via a linear program, which can be efficiently solved by the linprog function in MATLAB. Several cases were considered, such as $\left(P_{1},P_{2}\right)=\left(1,1\right),\left(P_{1},P_{2}\right)=\left(1,2\right)$, $\left(P_{1},P_{2}\right)=\left(3,1\right)$, etc. In all these cases, the numerical evaluation of (46) leads to the same value as the lower bound in (51). Since the problem is not convex, it is not known whether the numerical results are the global optimum solutions; hence, we leave it as a conjecture that the sum capacity can be achieved by time division with power control.

## 6. Conclusions

We have studied the capacity region of a two-transmitter Gaussian MAC under average input power constraints and one-bit ADC front end at the receiver. We have derived an upper bound on the cardinality of this auxiliary variable, and proved that the distributions that achieve the boundary points of the capacity region are finite and discrete. Finally, a lower bound is proposed on the sum capacity of this MAC that is achieved by time division with power control. Through numerical analysis, this lower bound is shown to be tight.

## Figures and Tables

**Figure A1:**
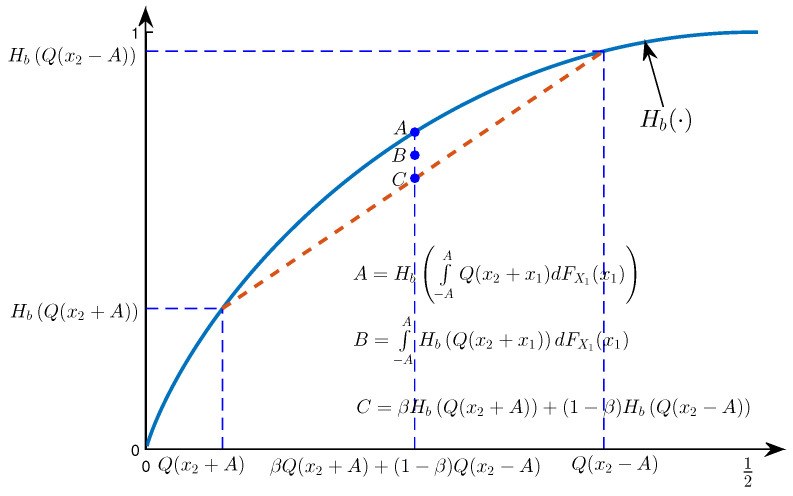
The figure depicting (A46) and (A48). Note that in the statement of Lemma 3, $x_{2}\to +\infty$. Hence, we have assumed $x_{2}>A$ in the figure.

## Appendix A

The capacity region of the discrete memoryless (DM) MAC with input cost constraints has been addressed in Exercise 4.8 of [10]. If the input alphabets are not discrete, the capacity region is still the same because: (1) the converse remains the same if the inputs are from a continuous alphabet; (2) the region is achievable by coded time sharing and the discretization procedure (see Remark 3.8 in [10]). Therefore, it is sufficient to show the cardinality bound |U|≤5.

Let P be the set of all product distributions (i.e., of the form $F_{X_{1}}\left(x_{1}\right)F_{X_{2}}\left(x_{2}\right)$) on $R^{2}$. Let $g:P\to R^{5}$ be a vector-valued mapping defined element-wise as:

(A1) $$\begin{array}{cc} g_{1}\left(F_{X_{1}|U}\left(\cdot |u\right)F_{X_{2}|U}\left(\cdot |u\right)\right) & =I(X_{1};Y|X_{2},U=u),\\ g_{2}\left(F_{X_{1}|U}\left(\cdot |u\right)F_{X_{2}|U}\left(\cdot |u\right)\right) & =I(X_{2};Y|X_{1},U=u),\\ g_{3}\left(F_{X_{1}|U}\left(\cdot |u\right)F_{X_{2}|U}\left(\cdot |u\right)\right) & =I(X_{1},X_{2};Y|U=u),\\ g_{4}\left(F_{X_{1}|U}\left(\cdot |u\right)F_{X_{2}|U}\left(\cdot |u\right)\right) & =E[X_{1}^{2}|U=u],\\ g_{5}\left(F_{X_{1}|U}\left(\cdot |u\right)F_{X_{2}|U}\left(\cdot |u\right)\right) & =E[X_{2}^{2}|U=u]. \end{array}$$

Let $G\subset R^{5}$ be the image of P under the mapping g (i.e., G=g(P)). Given an arbitrary $\left(U,X_{1},X_{2}\right)∼F_{U}F_{X_{1}|U}F_{X_{2}|U}$, we obtain the vector r as:

$$\begin{array}{cc} r_{1} & =I\left(X_{1};Y|X_{2},U\right)=\int_{U}I\left(X_{1};Y|X_{2},U=u\right)dF_{U}\left(u\right),\\ r_{2} & =I\left(X_{2};Y|X_{1},U\right)=\int_{U}I\left(X_{2};Y|X_{1},U=u\right)dF_{U}\left(u\right),\\ r_{3} & =I\left(X_{1},X_{2};Y|U\right)=\int_{U}I\left(X_{1},X_{2};Y|U=u\right)dF_{U}\left(u\right),\\ r_{4} & =E\left[X_{1}^{2}\right]=\int_{U}E\left[X_{1}^{2}|U=u\right]dF_{U}\left(u\right),\\ r_{5} & =E\left[X_{2}^{2}\right]=\int_{U}E\left[X_{2}^{2}|U=u\right]dF_{U}\left(u\right). \end{array}$$

Therefore, r is in the convex hull of $G\subset R^{5}$. By Carathéodory’s theorem [9], r can be written as a convex combination of six (=5+1) or fewer points in G, which states that it is sufficient to consider |U|≤6. Since P is a connected set (P is the product of two connected sets; therefore, it is connected. Each of the sets in this product is connected because of being a convex vector space.) and the mapping g is continuous (this is a direct result of the continuity of the channel transition probability), G is a connected subset of $R^{5}$. Therefore, the connectedness of G refines the cardinality of *U* to |U|≤5.

It is also important to note that for the boundary points of $C(P_{1},P_{2})$ that are not sum-rate optimal, it is sufficient to have |U|≤4. The proof is as follows. Any point on the boundary of the capacity region that does not maximize $R_{1}+R_{2}$ is either of the form $\left(I\left(X_{1};Y|X_{2},U\right),I\left(X_{2};Y|U\right)\right)$ or $\left(I\left(X_{1};Y|U\right),I\left(X_{2};Y|X_{1},U\right)\right)$ for some $F_{U}F_{X_{1}|U}F_{X_{2}|U}$ that satisfies $E\left[X_{j}^{2}\right]\leq P_{j},j=1,2.$ In other words, it is one of the corner points of the corresponding pentagon in (4). As in the proof of Proposition 1, define the mapping $g:P\to R^{4}$, where $g_{1}$ and $g_{2}$ are the coordinates of this boundary point conditioned on U=u, and $g_{3}$, $g_{4}$ are the same as $g_{4}$ and $g_{5}$ in (A1), respectively. The sufficiency of |U|≤4 in this case follows similarly.

## Appendix B

Since |U|≤5, we assume U={0,1,2,3,4} without loss of generality, since what matters in the evaluation of the capacity region is the mass probability of the auxiliary random variable *U*, not its actual values.

In order to show the compactness of Ω, we adopt a general form of the approach in [12].

First, we show that Ω is tight (a set of probability distributions Θ defined on $R^{k}$, i.e., the set of CDFs $F_{X_{1},X_{2},\ldots ,X_{k}}$, is said to be tight, if for every ϵ>0, there is a compact set $K_{\epsilon }\subset R^{k}$ such that [14]:

$$\mathrm{Pr}\left\{\left(X_{1},X_{2},\ldots ,X_{k}\right)\in R^{k}\backslash K_{\epsilon }\right\}<\epsilon ,\;\forall F_{X_{1},X_{2},\ldots ,X_{k}}\in \Theta .$$

Choose $T_{j}$, j=1,2, such that $T_{j}>\sqrt{\frac{2P_{j}}{\epsilon }}$. Then, from Chebyshev’s inequality,

(A2) $$\mathrm{Pr}\left\{|X_{j}|>T_{j}\right\}\leq \frac{P_{j}}{T_{j}^{2}}<\frac{\epsilon }{2},\;j=1,2.$$

Let $K_{\epsilon }=\left[0,4\right]\times \left[-T_{1},T_{1}\right]\times \left[-T_{2},T_{2}\right]\subset R^{3}$. It is obvious that $K_{\epsilon }$ is a closed and bounded subset of $R^{3}$ and, therefore, compact. With this choice of $K_{\epsilon }$, we have:

(A3) $$\begin{array}{cc} \mathrm{Pr}\left\{\left(U,X_{1},X_{2}\right)\in R^{3}\setminus K_{\epsilon }\right\} & \leq \mathrm{Pr}\left\{U\notin \left[0,4\right]\right\}+\mathrm{Pr}\left\{X_{1}\notin \left[-T_{1},T_{1}\right]\right\}+\mathrm{Pr}\left\{X_{2}\notin \left[-T_{2},T_{2}\right]\right\}\\ & <0+\frac{\epsilon }{2}+\frac{\epsilon }{2}=\epsilon , \end{array}$$

where (A3) is due to (A2). Hence, Ω is tight.

From Prokhorov’s theorem [14] (p. 318), a set of probability distributions is tight if and only if it is relatively sequentially compact (a subset of topological space is relatively compact if its closure is compact). This means that for every sequence of CDFs $\left\{F_{n}\right\}$ in Ω, there exists a subsequence $\left\{F_{n_{k}}\right\}$ that is weakly convergent (the weak convergence of $\left\{F_{n}\right\}$ to *F* (also shown as $F_{n}\left(x\right)\overset{w}{\to }F\left(x\right)$) is equivalent to:

(A4) $$\lim_{n\to \infty }\int_{R}\psi \left(x\right)dF_{n}\left(x\right)=\int_{R}\psi \left(x\right)dF\left(x\right),$$

for all continuous and bounded functions ψ(·) on R. Note that $F_{n}\left(x\right)\overset{w}{\to }F\left(x\right)$ if and only if $d_{L}\left(F_{n},F\right)\to \;0$.) to a CDF $F_{0}$, which is not necessarily in Ω. If we can show that this $F_{0}$ is also an element of Ω, then the proof is complete, since we have shown that Ω is sequentially compact and, therefore, compact (Compactness and sequential compactness are equivalent in metric spaces. Note that Ω is a metric space with Lévy distance.).

Assume a sequence of distributions $\left\{F_{n}\left(\cdot ,\cdot ,\cdot \right)\right\}$ in Ω that converges weakly to $F_{0}\left(\cdot ,\cdot ,\cdot \right)$. In order to show that this limiting distribution is also in Ω, we need to show that both the average power constraints and the Markov chain ($X_{1}-U-X_{2}$) are preserved under $F_{0}$. The preservation of the second moment follows similarly to the argument in (Appendix I, [12]). In other words, since $x^{2}$ is continuous and bounded from below, from Theorem 4.4.4 in [15]:

(A5) $$\begin{array}{cc} \int x_{j}^{2}d^{3}F_{0}\left(u,x_{1},x_{2}\right) & \leq \underset{n\to \infty }{lim inf}\int x_{j}^{2}d^{3}F_{n}\left(u,x_{1},x_{2}\right)\\ & \leq P_{j},\;j=1,2, \end{array}$$

Therefore, the second moments are preserved under the limiting distribution $F_{0}$.

For the preservation of the Markov chain $X_{1}-U-X_{2}$, we need the following proposition.

**Proposition** **A1.**
*Assume a sequence of distributions $\left\{F_{n}\left(\cdot ,\cdot \right)\right\}$ over the pair of random variables (X,Y) that converges weakly to $F_{0}\left(\cdot ,\cdot \right)$. Furthermore, assume that Y has a finite support, i.e., Y={1,2,…,|Y|}. Then, the sequence of conditional distributions (conditioned on Y) converges weakly to the limiting conditional distribution (conditioned on Y), i.e.,*

(A6) $$F_{n}\left(\cdot |y\right)\overset{w}{\to }F_{0}\left(\cdot |y\right),\;\forall y\in Y,p_{0}\left(y\right)>0.$$

**Proof** **of** **Proposition** **A1.** The proof is by contradiction. If (A6) is not true, then there exists $y^{\prime }\in Y$, such that $p_{0}\left(y^{\prime }\right)>0$ and Fn(·|y′)→wF0(·|y′). This means, from the definition of weak convergence, that there exists a bounded continuous function of *x*, denoted by $g_{y^{\prime }}\left(x\right)$, such that:

(A7) ∫gy′(x)dFn(x|y′)→∫gy′(x)dF0(x|y′).

Let f(x,y) be any bounded continuous function that satisfies:

(A8) $$f\left(x,y\right)=\left\{\begin{array}{cc} 0 & y\in Y,\;y\neq y^{\prime }\\ g_{y^{\prime }}\left(x\right) & y=y^{\prime } \end{array}\right..$$

With this choice of f(x,y), we have:

(A9) ∫f(x,y)d2Fn(x,y)→∫f(x,y)d2F0(x,y),

which violates the assumption of the weak convergence of $F_{n}\left(\cdot ,\cdot \right)$ to $F_{0}\left(\cdot ,\cdot \right)$. Therefore, (A6) holds. ☐

Since $\left\{F_{n}\left(\cdot ,\cdot ,\cdot \right)\right\}$ in Ω converges weakly to $F_{0}\left(\cdot ,\cdot ,\cdot \right)$ and U is finite, from Proposition A1, we have:

(A10) $$F_{n}\left(\cdot ,\cdot |u\right)\overset{w}{\to }F_{0}\left(\cdot ,\cdot |u\right),\;\forall u\in U,$$

where it is obvious that the arguments are $x_{1}$ and $x_{2}$. Since $F_{n}\in \Omega$, we have $F_{n}\left(x_{1},x_{2}|u\right)=F_{n}\left(x_{1}|u\right)F_{n}\left(x_{2}|u\right)\;\forall u\in U$. Furthermore, since the convergence of the joint distribution implies the convergence of the marginals, we have (Theorem 2.7, [16,17]),

(A11) $$F_{0}\left(x_{1},x_{2}|u\right)=F_{0}\left(x_{1}|u\right)F_{0}\left(x_{2}|u\right)\;\forall u\in U,$$

which states that under the limiting distribution $F_{0}$, the Markov chain $X_{1}-U-X_{2}$ is preserved (Alternatively, this could be proven by the lower-semicontinuity of the mutual information as follow:

(A12) $$\begin{array}{cc} I_{F_{0}}\left(X_{1};X_{2}|U=u\right) & \leq \underset{n\to \infty }{lim inf}I_{F_{n}}\left(X_{1};X_{2}|U=u\right) \end{array}$$

(A13) $$\begin{array}{c} =0,\;\forall u\in U, \end{array}$$

where $I_{F}$ denotes the mutual information under distribution *F*. The last equality is from the conditional independence of $X_{1}$ and $X_{2}$ given U=u under $F_{n}$. Therefore, $I_{F_{0}}\left(X_{1};X_{2}|U=u\right)=0,\;\forall u\in U$, which is equivalent to (A11).). This completes the proof of the compactness of Ω.

## Appendix C

### Appendix C.1. Concavity

When 0<λ≤1, we have:

(A14) $$I_{\lambda }\left(F_{X_{1}}F_{X_{2}}\right)=\lambda H\left(Y\right)+\left(1-\lambda \right)H\left(Y|X_{2}\right)-H\left(Y|X_{1},X_{2}\right).$$

For a given $F_{X_{1}}$, H(Y) is a concave function of $F_{X_{2}}$, while $H(Y|X_{2})$ and $H(Y|X_{1},X_{2})$ are linear in $F_{X_{2}}$. Therefore, $I_{\lambda }$ is a concave function of $F_{X_{2}}$. For a given $F_{X_{2}}$, H(Y) and $H(Y|X_{2})$ are concave functions of $F_{X_{1}}$, while $H(Y|X_{1},X_{2})$ is linear in $F_{X_{1}}$. Since (1−λ)≥0, $I_{\lambda }$ is a concave function of $F_{X_{1}}$. The same reasoning applies to the case λ>1.

### Appendix C.2. Continuity

When λ≤1, the continuity of the three terms on the RHS of (A14) is investigated. Let $\left\{F_{X_{2},n}\right\}$ be a sequence of distributions, which is weakly convergent to $F_{X_{2}}$. For a given $F_{X_{1}}$, we have:

(A15) $$\begin{array}{cc} \lim_{x_{2}\to x_{2}^{0}}p\left(y;F_{X_{1}}|x_{2}\right) & =\lim_{x_{2}\to x_{2}^{0}}\int Q\left(x_{1}+x_{2}\right)dF_{X_{1}}\left(x_{1}\right)\\ & =\int \lim_{x_{2}\to x_{2}^{0}}Q\left(x_{1}+x_{2}\right)dF_{X_{1}}\left(x_{1}\right) \end{array}$$

(A16) $$\begin{array}{c} =p(y;F_{X_{1}}|x_{2}^{0}), \end{array}$$

where (A15) is due to the fact that the *Q* function can be dominated by one, which is an absolutely integrable function over $F_{X_{1}}$. Therefore, $p(y;F_{X_{1}}|x_{2})$ is continuous in $x_{2}$, and combined with the weak convergence of $\left\{F_{X_{2},n}\right\}$, we can write:

$$\begin{array}{cc} \lim_{n\to \infty }p\left(y;F_{X_{1}}F_{X_{2},n}\right) & =\lim_{n\to \infty }\int p\left(y;F_{X_{1}}|x_{2}\right)dF_{X_{2},n}\left(x_{2}\right)\\ & =\int p\left(y;F_{X_{1}}|x_{2}\right)dF_{X_{2}}\left(x_{2}\right)\\ & =p(y;F_{X_{1}}F_{X_{2}}). \end{array}$$

This allows us to write:

$$\begin{array}{c} \lim_{n\to \infty }-\sum_{y=0}^{1}p\left(y;F_{X_{1}}F_{X_{2},n}\right)\log p\left(y;F_{X_{1}}F_{X_{2},n}\right)=-\sum_{y=0}^{1}p\left(y;F_{X_{1}}F_{X_{2}}\right)\log p\left(y;F_{X_{1}}F_{X_{2}}\right), \end{array}$$

which proves the continuity of H(Y) in $F_{X_{2}}$. $H(Y|X_{2}=x_{2})$ is a bounded (∈[0,1]) continuous function of $x_{2}$, since it is a continuous function of $p(y;F_{X_{1}}|x_{2})$, and the latter is continuous in $x_{2}$ (see (A16)). Therefore,

$$\lim_{n\to \infty }\int H\left(Y|X_{2}=x_{2}\right)dF_{X_{2},n}\left(x_{2}\right)=\int H\left(Y|X_{2}=x_{2}\right)dF_{X_{2}}\left(x_{2}\right),$$

which proves the continuity of $H(Y|X_{2})$ in $F_{X_{2}}$. In a similar way, it can be verified that $\int H\left(Y|X_{1}\;=\;x_{1},X_{2}=x_{2}\right)dF_{X_{1}}\left(x_{1}\right)$ is a bounded and continuous function of $x_{2}$, which guarantees the continuity of $H(Y|X_{1},X_{2})$ in $F_{X_{2}}$, since:

(A17) $$H\left(Y|X_{1},X_{2}\right)=\int \left(\int H\left(Y|X_{1}=x_{1},X_{2}=x_{2}\right)dF_{X_{1}}\left(x_{1}\right)\right)dF_{X_{2}}\left(x_{2}\right)$$

Therefore, for a given $F_{X_{1}}$, $I_{\lambda }$ is a continuous function of $F_{X_{2}}$. Exchanging the roles of $F_{X_{1}}$ and $F_{X_{2}}$, also the case λ>1 can be addressed similarly, so they are omitted for the sake of brevity.

### Appendix C.3. Weak Differentiability

For a given $F_{X_{1}}$, the weak derivative of $I_{\lambda }$ at $F_{X_{2}}^{0}$ is given by:

(A18) $$I_{\lambda }^{\prime }\left(F_{X_{1}}F_{X_{2}}\right){|}_{F_{X_{2}}^{0}}=\lim_{\beta \to 0^{+}}\frac{I_{\lambda }\left(F_{X_{1}}\left(\left(1-\beta \right)F_{X_{2}}^{0}+\beta F_{X_{2}}\right)\right)-I_{\lambda }\left(F_{X_{1}}F_{X_{2}}^{0}\right)}{\beta },$$

if the limit exists. It can be verified that:

$$\begin{array}{c} I_{\lambda }^{\prime }\left(F_{X_{1}}F_{X_{2}}\right){|}_{F_{X_{2}}^{0}}\\ =\lim_{\beta \to 0^{+}}\frac{\int i_{\lambda }\left(x_{2};\left(1-\beta \right)F_{X_{2}}^{0}+\beta F_{X_{2}}|F_{X_{1}}\right)d\left(\left(1-\beta \right)F_{X_{2}}^{0}\left(x_{2}\right)+\beta F_{X_{2}}\left(x_{2}\right)\right)-\int i_{\lambda }\left(x_{2};F_{X_{2}}^{0}|F_{X_{1}}\right)dF_{X_{2}}^{0}\left(x_{2}\right)}{\beta }\\ =\int i_{\lambda }\left(x_{2};F_{X_{2}}^{0}|F_{X_{1}}\right)dF_{X_{2}}\left(x_{2}\right)-\int i_{\lambda }\left(x_{2};F_{X_{2}}^{0}|F_{X_{1}}\right)dF_{X_{2}}^{0}\left(x_{2}\right)\\ =\int i_{\lambda }\left(x_{2};F_{X_{2}}^{0}|F_{X_{1}}\right)dF_{X_{2}}\left(x_{2}\right)-I_{\lambda }\left(F_{X_{1}}F_{X_{2}}^{0}\right), \end{array}$$

where $i_{\lambda }$ has been defined in (17). In a similar way, for a given $F_{X_{2}}$, the weak derivative of $I_{\lambda }$ at $F_{X_{1}}^{0}$ is:

(A19) $$I_{\lambda }^{\prime }\left(F_{X_{1}}F_{X_{2}}\right){|}_{F_{X_{1}}^{0}}=\int {\tilde{i}}_{\lambda }\left(x_{1};F_{X_{1}}^{0}|F_{X_{2}}\right)dF_{X_{1}}\left(x_{1}\right)-I_{\lambda }\left(F_{X_{1}}^{0}F_{X_{2}}\right),$$

where ${\tilde{i}}_{\lambda }$ has been defined in (16). The case λ>1 can be addressed similarly.

## Appendix D

We have:

(A20) $$\begin{array}{cc} \underset{\begin{array}{c} F_{X_{1}}:\\ E\left[X_{1}^{2}\right]\leq P_{1}^{\prime } \end{array}}{\sup}I_{\lambda }\left(F_{X_{1}}F_{X_{2}}\right) & \leq \underset{\begin{array}{c} F_{X_{1}}F_{X_{2}}:\\ E\left[X_{j}^{2}\right]\leq P_{j}^{\prime },\;j=1,2 \end{array}}{\sup}I_{\lambda }\left(F_{X_{1}}F_{X_{2}}\right)\\ & \leq \underset{\begin{array}{c} F_{X_{1}}F_{X_{2}}:\\ E\left[X_{j}^{2}\right]\leq P_{j}^{\prime },\;j=1,2 \end{array}}{\sup}I\left(X_{1},X_{2};Y\right) \end{array}$$

(A21) $$\begin{array}{c} \leq \underset{\begin{array}{c} F_{X_{1}}F_{X_{2}}:\\ E\left[X_{j}^{2}\right]\leq P_{j}^{\prime },\;j=1,2 \end{array}}{\sup}H\left(Y\right)-\underset{\begin{array}{c} F_{X_{1}}F_{X_{2}}:\\ E\left[X_{j}^{2}\right]\leq P_{j}^{\prime },\;j=1,2 \end{array}}{\inf}H\left(Y|X_{1},X_{2}\right)\\ =1-\underset{\begin{array}{c} F_{X_{1}}F_{X_{2}}:\\ E\left[X_{j}^{2}\right]\leq P_{j}^{\prime },\;j=1,2 \end{array}}{\inf}\int \;\;\int H_{b}\left(Q(x_{1}+x_{2})\right)dF_{X_{1}}\left(x_{1}\right)dF_{X_{2}}\left(x_{2}\right)\\ =1-\underset{\begin{array}{c} F_{X_{1}}F_{X_{2}}:\\ E\left[X_{j}^{2}\right]\leq P_{j}^{\prime },\;j=1,2 \end{array}}{\inf}\int \;\;\int H_{b}\left(Q\left(\sqrt{x_{1}^{2}}+\sqrt{x_{2}^{2}}\right)\right)dF_{X_{1}}\left(x_{1}\right)dF_{X_{2}}\left(x_{2}\right) \end{array}$$

(A22) $$\begin{array}{c} \leq 1-\underset{\begin{array}{c} F_{X_{1}}F_{X_{2}}:\\ E\left[X_{j}^{2}\right]\leq P_{j}^{\prime },\;j=1,2 \end{array}}{\inf}\int \;\;\int Q\left(\sqrt{x_{1}^{2}}+\sqrt{x_{2}^{2}}\right)dF_{X_{1}}\left(x_{1}\right)dF_{X_{2}}\left(x_{2}\right) \end{array}$$

(A23) $$\begin{array}{c} =1-Q\left(\sqrt{P_{1}^{\prime }}+\sqrt{P_{2}^{\prime }}\right) \end{array}$$

(A24) $$\begin{array}{c} <1, \end{array}$$

where (A20) is from the non-negativity of mutual information and the assumption that 0<λ≤1; (A21) is justified since the *Q* function is monotonically decreasing and the sign of the inputs does not affect the average power constraints; $X_{1}$ and $X_{2}$ can be assumed non-negative (or alternatively non-positive) without loss of optimality; in (A22), we use the fact that $Q\left(\sqrt{x_{1}^{2}}+\sqrt{x_{2}^{2}}\right)\leq \frac{1}{2}$, and for $t\in [0,\frac{1}{2}]$, $H_{b}\left(t\right)\geq t$; (A23) is based on the convexity and monotonicity of the function $Q(\sqrt{u}+\sqrt{v})$ in (u,v), which is shown in Appendix G. Therefore, the LHS of (13) is strictly less than one.

Since $X_{2}$ has a finite second moment ($E\left[X_{2}^{2}\right]\leq P_{2}^{\prime }$), from Chebyshev’s inequality, we have:

(A25) $$P(|X_{2}\left|\geq M\right)\leq \frac{P_{2}^{\prime }}{M^{2}},\;\;\forall M>0.$$

Fix M>0, and consider $X_{1}∼F_{X_{1}}\left(x_{1}\right)=\frac{1}{2}\left[s\left(x_{1}+2M\right)+s\left(x_{1}-2M\right)\right]$. By this choice of $F_{X_{1}}$, we get:

(A26) $$\begin{array}{cc} I_{\lambda }\left(F_{X_{1}}F_{X_{2}}\right) & =I\left(X_{1};Y|X_{2}\right)+\lambda I\left(X_{2};Y\right)\\ & \geq I(X_{1};Y|X_{2})\\ & =\int_{-\infty }^{+\infty }I\left(X_{1};Y|X_{2}=x_{2}\right)dF_{X_{2}}\left(x_{2}\right)\\ & \geq \int_{-M}^{+M}I\left(X_{1};Y|X_{2}=x_{2}\right)dF_{X_{2}}\left(x_{2}\right)\\ & \geq \underset{F_{X_{2}}}{\inf}\int_{-M}^{+M}H\left(Y|X_{2}=x_{2}\right)dF_{X_{2}}\left(x_{2}\right)-\underset{F_{X_{2}}}{\sup}\int_{-M}^{+M}H\left(Y|X_{1},X_{2}=x_{2}\right)dF_{X_{2}}\left(x_{2}\right) \end{array}$$

(A27) $$\begin{array}{c} \geq \left(1-\frac{P_{2}^{\prime }}{M^{2}}\right)H_{b}\left(\frac{1}{2}-\frac{1}{2}\left(Q\left(3M\right)+Q\left(M\right)\right)\right)-H_{b}\left(Q(2M)\right), \end{array}$$

where (A27) is due to (A25) and the fact that $H\left(Y|X_{2}=x_{2}\right)=H_{b}\left(\frac{1}{2}Q\left(2M+x_{2}\right)+\frac{1}{2}Q\left(-2M+x_{2}\right)\right)$ is minimized over [−M,M] at $x_{2}=M$ (or, alternatively at $x_{2}=-M$) and $H\left(Y|X_{1},X_{2}=x_{2}\right)=\frac{1}{2}H_{b}\left(Q\left(2M+x_{2}\right)\right)+\frac{1}{2}H_{b}\left(Q\left(-2M+x_{2}\right)\right)$ is maximized at $x_{2}=0$. (A27) shows that $I_{\lambda }$ (≤1) can become arbitrarily close to one given that *M* is large enough. Hence, its supremum over all distributions $F_{X_{1}}$ is one. This means that (13) cannot hold, and $\theta_{1}\neq 0$.

## Appendix E. Justification of (18), (19) and (20)

Let *X* be a vector space and *Z* be a real-valued function defined on a convex domain D⊂X. Suppose that $x^{*}$ maximizes *Z* on *D* and that *Z* is Gateaux differentiable (weakly differentiable) at $x^{*}$. Then, from (Theorem 2, p. 178, [18]),

(A28) $$Z^{\prime }{\left(x\right)|}_{x^{*}}\leq 0,$$

where $Z^{\prime }{\left(x\right)|}_{x^{*}}$ is the weak derivative of *Z* at $x^{*}$.

From (A19), we have the weak derivative of $I_{\lambda }$ at $F_{X_{1}}^{*}$ as:

(A29) $$I_{\lambda }^{\prime }\left(F_{X_{1}}F_{X_{2}}\right){|}_{F_{X_{1}}^{*}}=\int {\tilde{i}}_{\lambda }\left(x_{1};F_{X_{1}}^{*}|F_{X_{2}}\right)dF_{X_{1}}\left(x_{1}\right)-I_{\lambda }\left(F_{X_{1}}^{*}F_{X_{2}}\right).$$

Now, the derivation of (18) is immediate by inspecting that the weak derivative of the objective of (11) at $F_{X_{1}}^{*}$ is given by:

(A30) $$\begin{array}{cc} I_{\lambda }^{\prime }\left(F_{X_{1}}F_{X_{2}}\right){|}_{F_{X_{1}}^{*}}-\theta_{1}\left(\int x_{1}^{2}dF_{X_{1}}\left(x_{1}\right)-\int x_{1}^{2}dF_{X_{1}}^{*}\left(x_{1}\right)\right) & =\int {\tilde{i}}_{\lambda }\left(x_{1};F_{X_{1}}^{*}|F_{X_{2}}\right)dF_{X_{1}}\left(x_{1}\right)-I_{\lambda }\left(F_{X_{1}}^{*}F_{X_{2}}\right)\\ & -\theta_{1}\left(\int x_{1}^{2}dF_{X_{1}}\left(x_{1}\right)-\int x_{1}^{2}dF_{X_{1}}^{*}\left(x_{1}\right)\right). \end{array}$$

Letting (A30) be lower than or equal to zero (as in (A28)) results in (18).

The equivalence of (18) to (19) and (20) follows similarly to the proof of Corollary 1 in (p. 210, [19]).

## Appendix F

Equation (21) is obtained as follows.

(A31) $$\begin{array}{cc} \left|D\left(p\left(y|x_{1},x_{2}\right)\left|\right|p\left(y;F_{X_{1}}F_{X_{2}}\right)\right)\right| & =\left|\sum_{y=0}^{1}p\left(y|x_{1},x_{2}\right)\log\frac{p(y|x_{1},x_{2})}{p(y;F_{X_{1}}F_{X_{2}})}\right|\\ & \leq |H\left(Y|X_{1}=x_{1},X_{2}=x_{2}\right)|+\left|\sum_{y=0}^{1}p\left(y|x_{1},x_{2}\right)\log p\left(y;F_{X_{1}}F_{X_{2}}\right)\right|\\ & \leq 1+\left|\sum_{y=0}^{1}\log p\left(y;F_{X_{1}}F_{X_{2}}\right)\right| \end{array}$$

(A32) $$\begin{array}{c} =1-\sum_{y=0}^{1}\log p\left(y;F_{X_{1}}F_{X_{2}}\right)\\ \leq 1-2\min\left\{\log p_{Y}\left(0;F_{X_{1}}F_{X_{2}}\right),\log p_{Y}\left(1;F_{X_{1}}F_{X_{2}}\right)\right\}\\ \leq 1-2\log Q(\sqrt{P_{1}^{\prime }}+\sqrt{P_{2}^{\prime }})\\ <\infty , \end{array}$$

where (A31) is due to the fact that the binary entropy function is upper bounded by one. (A32) is justified as follows.

(A33) $$\begin{array}{cc} \min\left\{p_{Y}\left(0;F_{X_{1}}F_{X_{2}}\right),p_{Y}\left(1;F_{X_{1}}F_{X_{2}}\right)\right\} & \geq \underset{\begin{array}{c} F_{X_{1}}F_{X_{2}}:\\ E\left[X_{j}^{2}\right]\leq P_{j}^{\prime } \end{array}}{\inf}\min\left\{p_{Y}\left(0;F_{X_{1}}F_{X_{2}}\right),p_{Y}\left(1;F_{X_{1}}F_{X_{2}}\right)\right\}\\ & =\underset{\begin{array}{c} F_{X_{1}}F_{X_{2}}:\\ E\left[X_{j}^{2}\right]\leq P_{j}^{\prime } \end{array}}{\inf}p_{Y}\left(0;F_{X_{1}}F_{X_{2}}\right)\\ & =\underset{\begin{array}{c} F_{X_{1}}F_{X_{2}}:\\ E\left[X_{j}^{2}\right]\leq P_{j}^{\prime } \end{array}}{\inf}\int \;\;\int Q\left(x_{1}+x_{2}\right)dF_{X_{1}}\left(x_{1}\right)dF_{X_{2}}\left(x_{2}\right)\\ & =\underset{\begin{array}{c} F_{X_{1}}F_{X_{2}}:\\ E\left[X_{j}^{2}\right]\leq P_{j}^{\prime } \end{array}}{\inf}\int \;\;\int Q\left(\sqrt{x_{1}^{2}}+\sqrt{x_{2}^{2}}\right)dF_{X_{1}}\left(x_{1}\right)dF_{X_{2}}\left(x_{2}\right) \end{array}$$

(A34) $$\begin{array}{c} \geq Q\left(\sqrt{P_{1}^{\prime }}+\sqrt{P_{2}^{\prime }}\right), \end{array}$$

where (A34) is based on the convexity and monotonicity of the function $Q(\sqrt{u}+\sqrt{v})$, which is shown in Appendix G.

Equation (22) is obtained as follows.

(A35) $$\begin{array}{cc} p(y;F_{X_{1}}|x_{2}) & \geq \min\left\{p\left(0;F_{X_{1}}|x_{2}\right),p\left(1;F_{X_{1}}|x_{2}\right)\right\}\\ & \geq \int Q\left(|x_{1}\left|+\right|x_{2}|\right)dF_{X_{1}}\left(x_{1}\right)\\ & =\int Q\left(\sqrt{x_{1}^{2}}+\left|x_{2}\right|\right)dF_{X_{1}}\left(x_{1}\right)\\ & \geq Q\left(\sqrt{P_{1}^{\prime }}+\left|x_{2}\right|\right), \end{array}$$

where (A35) is due to the convexity of $Q(\alpha +\sqrt{x})$ in *x* for α≥0.

Equation (23) is obtained as follows.

(A36) $$\begin{array}{cc} \left|\sum_{y=0}^{1}p\left(y|x_{1},x_{2}\right)\log\frac{p(y;F_{X_{1}}F_{X_{2}})}{p(y;F_{X_{1}}|x_{2})}\right| & \leq -\sum_{y=0}^{1}p\left(y|x_{1},x_{2}\right)\log p\left(y;F_{X_{1}}|x_{2}\right)-\sum_{y=0}^{1}p\left(y|x_{1},x_{2}\right)\log p\left(y;F_{X_{1}}F_{X_{2}}\right)\\ \; & \leq -\sum_{y=0}^{1}\log p\left(y;F_{X_{1}}|x_{2}\right)-\sum_{y=0}^{1}\log p\left(y;F_{X_{1}}F_{X_{2}}\right) \end{array}$$

(A37) $$\begin{array}{c} \leq -2\log Q\left(\sqrt{P_{1}^{\prime }}+\sqrt{P_{2}^{\prime }}\right)-2\log Q\left(\sqrt{P_{1}^{\prime }}+\left|x_{2}\right|\right), \end{array}$$

where (A36) is from $p(y|x_{1},x_{2})\leq 1$; and (A37) is from (A35) and (A34).

Note that (A37) is integrable with respect to $F_{X_{2}}$ due to the concavity of $-\log Q(\alpha +\sqrt{x})$ in *x* for α≥0 as shown in Appendix G. In other words,

(A38) $$\begin{array}{cc} \int_{-\infty }^{+\infty }\left(-2\log Q\left(\sqrt{P_{1}^{\prime }}+\sqrt{P_{2}^{\prime }}\right)-2\log Q\left(\sqrt{P_{1}^{\prime }}+\left|x_{2}\right|\right)\right)dF_{X_{2}}\left(x_{2}\right) & <-4\log Q\left(\sqrt{P_{1}^{\prime }}+\sqrt{P_{2}^{\prime }}\right) \end{array}$$

(A39) $$\begin{array}{cc} \; & <+\infty . \end{array}$$

## Appendix G. Two Convex Functions

Let $f\left(x\right)=\log Q\left(a+\sqrt{x}\right)$ for x,a≥0. We have,

$$f^{\prime }\left(x\right)=-\frac{e^{-\frac{{\left(a+\sqrt{x}\right)}^{2}}{2}}}{2\sqrt{2\pi x}Q\left(a+\sqrt{x}\right)},$$

and:

(A40) $$f^{\prime \prime }\left(x\right)=\frac{e^{-\frac{{\left(a+\sqrt{x}\right)}^{2}}{2}}}{4x\sqrt{2\pi }Q^{2}\left(a+\sqrt{x}\right)}\left(\left(a+\sqrt{x}+\frac{1}{\sqrt{x}}\right)Q\left(a+\sqrt{x}\right)-\phi \left(a+\sqrt{x}\right)\right),$$

where $\phi \left(x\right)=\frac{1}{\sqrt{2\pi }}e^{-\frac{x^{2}}{2}}$. Note that:

(A41) $$\begin{array}{cc} \left(1+at+t^{2}\right)Q\left(a+t\right)+a\phi \left(a+t\right) & >\left(1+{\left(a+t\right)}^{2}\right)Q\left(a+t\right) \end{array}$$

(A42) $$\begin{array}{c} >(a+t)\phi (a+t),\;\;\forall a,t>0, \end{array}$$

where (A41) and (A42) are, respectively, due to ϕ(x)>xQ(x) and $\left(1+x^{2}\right)Q\left(x\right)>x\phi \left(x\right)$ (x>0). Therefore,

$$\left(a+\sqrt{x}+\frac{1}{\sqrt{x}}\right)Q\left(a+\sqrt{x}\right)>\phi \left(a+\sqrt{x}\right),$$

which makes the second derivative in (A40) positive and proves the (strict) convexity of f(x).

Let $f\left(u,v\right)=Q\left(\sqrt{u}+\sqrt{v}\right)$ for u,v≥0. By simple differentiation, the Hessian matrix of *f* is:

(A43) $$H=\frac{e^{-\frac{{\left(\sqrt{u}+\sqrt{v}\right)}^{2}}{2}}}{\sqrt{2\pi }}\left[\begin{array}{cc} \frac{1}{2u\sqrt{u}}+\frac{\sqrt{u}+\sqrt{v}}{4u} & \frac{\sqrt{u}+\sqrt{v}}{4\sqrt{u}\sqrt{v}}\\ \frac{\sqrt{u}+\sqrt{v}}{4\sqrt{u}\sqrt{v}} & \frac{1}{2v\sqrt{v}}+\frac{\sqrt{u}+\sqrt{v}}{4v} \end{array}\right].$$

It can be verified that det(H)>0 and trace(H)>0. Therefore, both eigenvalues of H are positive, which makes the matrix positive definite. Hence, $Q(\sqrt{u}+\sqrt{v})$ is (strictly) convex in (u,v).

## Appendix H

Let $A≜\max\{A_{1},A_{2}\}$.

It is obvious that:

(A44) $$Q\left(x_{2}+A\right)\leq \int_{-A}^{A}Q\left(x_{1}+x_{2}\right)dF_{X_{1}}\left(x_{1}\right)\leq Q\left(x_{2}-A\right).$$

Therefore, we can write:

(A45) $$\int_{-A}^{A}Q\left(x_{1}+x_{2}\right)dF_{X_{1}}\left(x_{1}\right)=\beta Q\left(x_{2}+A\right)+\left(1-\beta \right)Q\left(x_{2}-A\right),$$

for some β∈[0,1]. Note that β is a function of $x_{2}$. Furthermore, due to the concavity of $H_{b}\left(\cdot \right)$, we have:

(A46) $$\begin{array}{cc} H_{b}\left(\int_{-A}^{A}Q\left(x_{1}+x_{2}\right)dF_{X_{1}}\left(x_{1}\right)\right) & \geq \int_{-A}^{A}H_{b}\left(Q\left(x_{1}+x_{2}\right)\right)dF_{X_{1}}\left(x_{1}\right). \end{array}$$

From the fact that:

(A47) $$H_{b}\left(x\right)\geq \frac{H_{b}\left(p\right)-H_{b}\left(a\right)}{p-a}\left(x-a\right)+H_{b}\left(a\right),\;\forall x\in \left[a,\;p\right],\;\forall a,p\in \left[0,\;1\right]\left(a<p\right),$$

we can also write:

(A48) $$\begin{array}{cc} \int_{-A}^{A}H_{b}\left(Q\left(x_{1}+x_{2}\right)\right)dF_{X_{1}}\left(x_{1}\right) & \geq \frac{H_{b}\left(Q\left(x_{2}-A\right)\right)\;-\;H_{b}\left(Q\left(x_{2}+A\right)\right)}{Q\left(x_{2}-A\right)\;-\;Q\left(x_{2}+A\right)}\left(\int_{-A}^{A}Q\left(x_{1}+x_{2}\right)dF_{X_{1}}\left(x_{1}\right)\;-\;Q\left(x_{2}+A\right)\right)\\ \; & \;\;\;+\;H_{b}\left(Q\left(x_{2}+A\right)\right)\\ & =\beta H_{b}\left(Q\left(x_{2}+A\right)\right)+\left(1-\beta \right)H_{b}\left(Q\left(x_{2}-A\right)\right), \end{array}$$

where (A45) and (A47) have been used in (A48). (A46) and (A48) are depicted in Figure A1.

From (A45) and (A48), we have:

(A49) $$\frac{\beta H_{b}\left(Q\left(x_{2}+A\right)\right)+\left(1-\beta \right)H_{b}\left(Q\left(x_{2}-A\right)\right)}{H_{b}\left(\beta Q\left(x_{2}+A\right)+\left(1-\beta \right)Q\left(x_{2}-A\right)\right)}\leq \frac{\int_{-A}^{A}H_{b}\left(Q\left(x_{1}+x_{2}\right)\right)dF_{X_{1}}\left(x_{1}\right)}{H_{b}\left(\int_{-A}^{A}Q\left(x_{1}+x_{2}\right)dF_{X_{1}}\left(x_{1}\right)\right)}\leq 1.$$

Let:

(A50) $$\beta^{*}≜\underset{\beta }{arg min}\frac{\beta H_{b}\left(Q\left(x_{2}+A\right)\right)+\left(1-\beta \right)H_{b}\left(Q\left(x_{2}-A\right)\right)}{H_{b}\left(\beta Q\left(x_{2}+A\right)+\left(1-\beta \right)Q\left(x_{2}-A\right)\right)}.$$

This minimizer satisfies the following equality:

(A51) $$\frac{d}{d\beta }\left(\frac{\beta H_{b}\left(Q\left(x_{2}+A\right)\right)+\left(1-\beta \right)H_{b}\left(Q\left(x_{2}-A\right)\right)}{H_{b}\left(\beta Q\left(x_{2}+A\right)+\left(1-\beta \right)Q\left(x_{2}-A\right)\right)}\right){|}_{\beta =\beta^{*}}=0.$$

Therefore, we can write:

(A52) $$\begin{array}{cc} \frac{\beta H_{b}\left(Q\left(x_{2}+A\right)\right)+\left(1-\beta \right)H_{b}\left(Q\left(x_{2}-A\right)\right)}{H_{b}\left(\beta Q\left(x_{2}+A\right)+\left(1-\beta \right)Q\left(x_{2}-A\right)\right)} & \geq \frac{\beta^{*}H_{b}\left(Q\left(x_{2}+A\right)\right)+\left(1-\beta^{*}\right)H_{b}\left(Q\left(x_{2}-A\right)\right)}{H_{b}\left(\beta^{*}Q\left(x_{2}+A\right)+\left(1-\beta^{*}\right)Q\left(x_{2}-A\right)\right)} \end{array}$$

(A53) $$\begin{array}{c} =\frac{\frac{H_{b}\left(Q\left(x_{2}-A\right)\right)-H_{b}\left(Q\left(x_{2}+A\right)\right)}{Q\left(x_{2}-A\right)-Q\left(x_{2}+A\right)}}{H_{b}^{\prime }\left(\beta^{*}Q\left(x_{2}+A\right)+\left(1-\beta^{*}\right)Q\left(x_{2}-A\right)\right)} \end{array}$$

(A54) $$\begin{array}{c} \geq \frac{\frac{H_{b}\left(Q\left(x_{2}-A\right)\right)-H_{b}\left(Q\left(x_{2}+A\right)\right)}{Q\left(x_{2}-A\right)-Q\left(x_{2}+A\right)}}{H_{b}^{\prime }\left(Q\left(x_{2}+A\right)\right)}, \end{array}$$

where (A52) is from the definition in (A50); (A53) is from the expansion of (A51), and $H_{b}^{\prime }\left(t\right)=\log\left(\frac{1-t}{t}\right)$ is the derivative of the binary entropy function; (A54) is due to the fact that $H_{b}^{\prime }\left(t\right)$ is a decreasing function.

Applying L’Hôspital’s rule multiple times, we obtain:

(A55) $$\begin{array}{cc} \lim_{x_{2}\to +\infty }\frac{\frac{H_{b}\left(Q\left(x_{2}-A\right)\right)-H_{b}\left(Q\left(x_{2}+A\right)\right)}{Q\left(x_{2}-A\right)-Q\left(x_{2}+A\right)}}{H_{b}^{\prime }\left(Q\left(x_{2}+A\right)\right)} & =\lim_{x_{2}\to +\infty }\frac{H_{b}\left(Q\left(x_{2}-A\right)\right)\left(1-\frac{H_{b}\left(Q\left(x_{2}+A\right)\right)}{H_{b}\left(Q\left(x_{2}-A\right)\right)}\right)}{Q\left(x_{2}-A\right)\left(1-\frac{Q(x_{2}+A)}{Q(x_{2}-A)}\right)\log\left(\frac{1-Q(x_{2}+A)}{Q(x_{2}+A)}\right)}\\ & =\lim_{x_{2}\to +\infty }-\frac{H_{b}\left(Q\left(x_{2}-A\right)\right)}{Q\left(x_{2}-A\right)\log\left(Q\left(x_{2}+A\right)\right)}\\ & =\lim_{x_{2}\to +\infty }\frac{e^{-\frac{{\left(x_{2}-A\right)}^{2}}{2}}\log\left(Q\left(x_{2}-A\right)\right)}{e^{-\frac{{\left(x_{2}-A\right)}^{2}}{2}}\log\left(Q\left(x_{2}+A\right)\right)+\frac{Q(x_{2}-A)}{Q(x_{2}+A)}e^{-\frac{{\left(x_{2}+A\right)}^{2}}{2}}}\\ & =\lim_{x_{2}\to +\infty }\frac{\log(Q\left(x_{2}-A\right))}{\log(Q\left(x_{2}+A\right))+1}\\ & =\lim_{x_{2}\to +\infty }\frac{Q\left(x_{2}+A\right)e^{Ax_{2}}}{Q\left(x_{2}-A\right)e^{-Ax_{2}}}\\ & =1 \end{array}$$

From (A49), (A54) and (A55), (35) is proven. Note that the boundedness of $X_{1}$ is crucial in the proof. In other words, the fact that $Q(x_{2}-A)\to 0$ as $x_{2}\to +\infty$ is the very result of A<+∞.
